# Focused allergic rhinitis practice parameter for Canada

**DOI:** 10.1186/s13223-024-00899-3

**Published:** 2024-08-08

**Authors:** Anne K. Ellis, Victoria Cook, Paul K. Keith, Sean R. Mace, William Moote, Andrew O’Keefe, Jaclyn Quirt, Lana Rosenfield, Peter Small, Wade Watson

**Affiliations:** 1 Division of Allergy & Immunology, Department of Medicine, Queen’s University, Kingston, ON Canada; 2 Community Allergy Clinic, Victoria, BC, and Department of Pediatrics, University of British Columbia, Vancouver, BC Canada; 3 Division of Clinical Immunology and Allergy, Department of Medicine, McMaster University, Hamilton, ON Canada; 4 Mace Allergy and Clinical Immunology, Toronto, ON Canada; 5 Western University, London, ON Canada; 6 Department of Pediatrics, Memorial University, St. John’s, NL Canada; 7 Department of Internal Medicine, University of Manitoba, Winnipeg, MB Canada; 8 Jewish General Hospital, Montreal, QC Canada; 9 Department of Pediatrics, Dalhousie University, Halifax, NS Canada

## Abstract

Allergic rhinitis (AR) is a prevalent disease in Canada that affects both children and adults. Several guidelines for the management of AR have been published by professional allergy societies worldwide. However, there are regional differences in the clinical management of AR, and regulatory approval of some AR pharmacotherapies varies among countries. Thus, six research questions specific to the treatment of AR in Canada were identified for this focused practice parameter. Reviews of the literature published since 2016 were conducted to obtain evidence-based support for the responses of the Work Group to each research question. In response to research question 1 “In patients with symptoms indicative of AR, is serum-specific IgE sufficient to identify candidates for immunotherapy or is a skin prick test mandatory?” the Work Group concluded that either sIgE testing or skin prick test are acceptable for diagnosing AR and guiding immunotherapy. In response to research question 2 “When taking into account the preferences of the patient and the prescriber (stakeholder engagement) should second-generation oral antihistamine (OAH) or intranasal corticosteroid (INCS) be first line?” the Work Group concluded that existing guidelines generally agree on the use of INCS as a first-line therapy used for AR, however, patient and provider preferences and considerations can easily shift the first choice to a second-generation OAH. In response to research question 3 “Is a combination intranasal antihistamine (INAH)/INCS formulation superior to INCS plus OAH? Do they become equivalent after prolonged use?” the Work Group concluded that that the combination INAH/INCS is superior to an INCS plus OAH. However, there was insufficient evidence to answer the second question. In response to research question 4 “Do leukotriene receptor antagonists (LTRA) have a greater benefit than OAH in AR for some symptoms to justify a therapeutic trial in those who cannot tolerate INCS?” the Work Group concluded that LTRAs have inferior, or at best equivalent, daytime or overall symptom control compared with OAH, but LTRAs may improve nighttime symptom control and provide benefits in patients with AR and concomitant asthma. In response to research question 5 “Should sublingual immunotherapy (SLIT) tablets be considered first-line immunotherapeutic options over subcutaneous immunotherapy (SCIT) based on the evidence of efficacy?” the Work Group concluded that the choice of SLIT or SCIT cannot be made on efficacy alone, and differences in other factors outweigh any differences in efficacy. In response to research question 6 “Based on efficacy data, should ALL patients seen by an allergist be offered SLIT or SCIT as a treatment option?” the Work Group concluded that the efficacy data suggests that SLIT or SCIT should be used broadly in patients with AR, but other clinical concerns also need to be taken into consideration.

**Supplementary Information:**

The online version contains supplementary material available at 10.1186/s13223-024-00899-3.

## Foreword

**Fig. 1:**
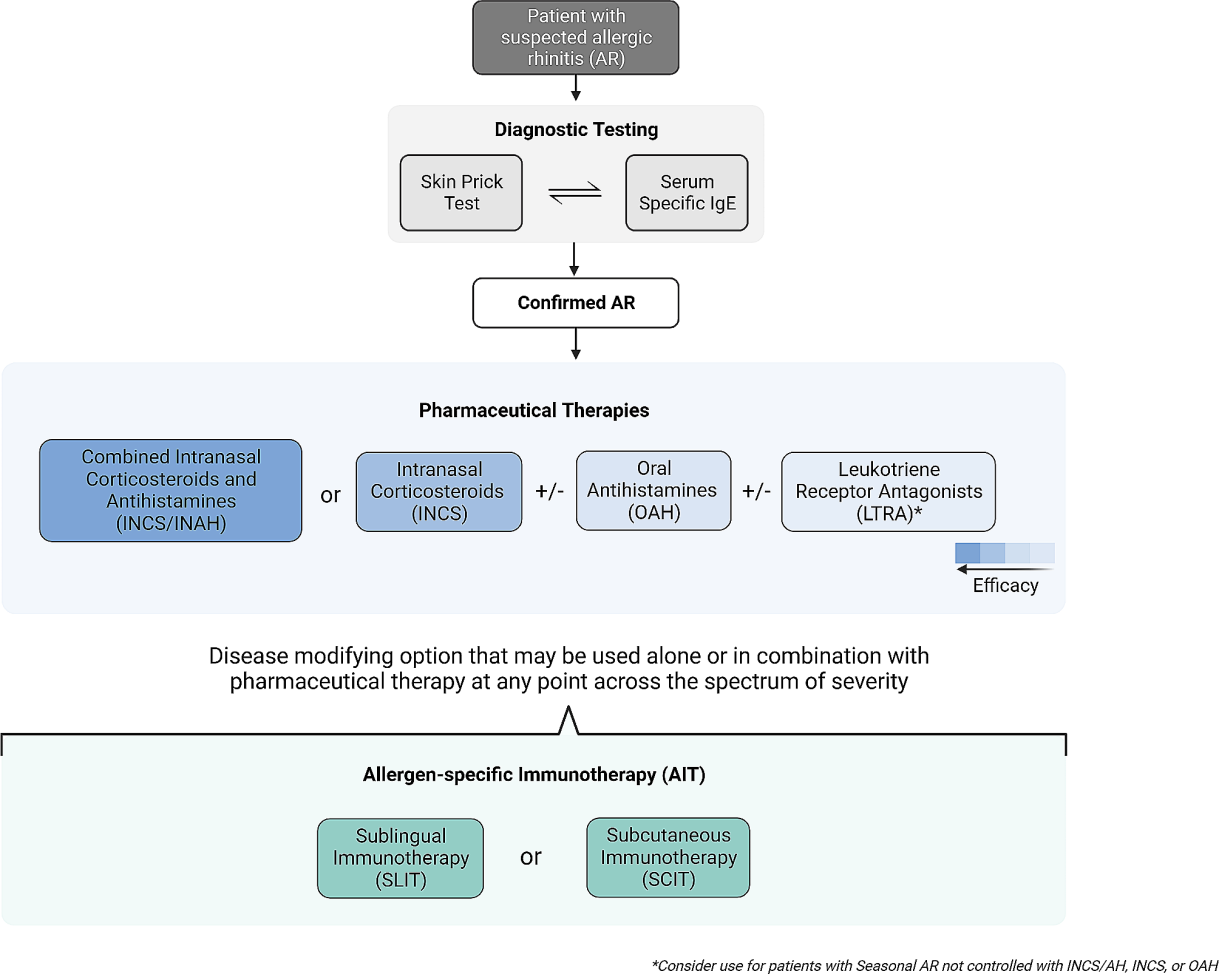
A stepwise algorithm for the diagnosis and treatment of allergic rhinitis. The choice of therapeutic intervention should be a shared decision-making process involving both the patient and the prescriber, with importance placed on balancing factors such as disease severity, therapy safety, and cost

Allergic rhinitis (AR) is a prevalent disease that affects both children and adults. In Canada, up to 20% of the general population is estimated to have a diagnosis of AR [1, 2]. The burden of AR is well documented, negatively impacting daily activities, sleep, work and school performance, and overall health-related quality of life. To reduce the burden of AR, disease management includes allergen avoidance, allergen immunotherapy (AIT), and symptom-relieving pharmacotherapies (Fig. 1) [3]. Several guidelines for the management of AR have been published by professional allergy societies worldwide. In the North American context, comprehensive, evidence-based practice parameters for seasonal AR (SAR) and rhinitis were updated in 2017 and 2020, respectively [4, 5]. Nevertheless, there are regional differences in the clinical management of AR, and regulatory approval of some AR pharmacotherapies varies among countries. Thus, research questions specific to the treatment of AR in Canada were identified by consensus of the Work Group and were chosen for this focused practice parameter. The 6 questions selected for the focused practice parameter were chosen due to the prevalence of unanswered questions and/or unmet needs from previous practice statements.

A GRADE approach was not used for the focused practice parameter because of the rarity of randomized control trial literature for some of the research questions. However, systematic reviews of the published literature from the past 5 years were conducted to obtain evidence-based support for the responses of the Work Group to each research question. Pharmacoeconomic analyses were not considered or conducted for the practice parameter, although the Work Group recognizes that economic factors can influence treatment decisions. We refer the reader to some reviews of the pharmacoeconomics for AR or cost-effectiveness among treatment options [6–8]. 

### Methods and overview of the practice parameter development process

A detailed and thorough literature search for appropriate medical literature in key sources was conducted for each of the 6 questions in a systematic manner by an information specialist. These sources included Ovid Medline database, Cochrane Database of Systematic Reviews, Cochrane Central Register of Controlled Trials, CMA Infobase, NICE guidelines database, Health Canada, National Institutes of Health, American Academy of Allergy, Asthma & Immunology, European Academy of Allergy and Clinical Immunology, Australasian Society of Clinical Immunology and Allergy, and the British Society for Allergy & Clinical Immunology. Advanced searching in Google was also conducted. Search strategies for each database and search terms for each internet resource for each of the research questions are described in Appendices 1–6, respectively. All the searches were limited to English only, human subject studies published in the year 2016 or later and included both pediatric and adult patients. The search results were screened for relevance to the research question, and specific inclusion and exclusion criteria for each research question were applied (see Appendices 1–6).

The searches focused on identifying high quality clinical practice guidelines, systematic reviews, and randomized controlled trials. However, other types of original research (e.g., cohort studies, retrospective studies, observational studies, etc.) were included in the search results for research questions 1, 3 and 4.

To identify the highest quality literature available on this topic, each unique clinical practice guideline, systematic review, and randomized controlled trial identified in each of the literature searches were appraised for quality. Each of these types of literature was appraised with a modified version of an appropriate framework.

Clinical practice guidelines were appraised with a modified AGREE II framework with a focus on two criteria: [9]

- Systematic methods were used to search for evidence.
- There is an explicit link between the recommendations and the supporting evidence.

Systematic reviews were appraised with a modified AMSTAR framework with a focus on two criteria: [10]

- Was a comprehensive literature search performed?
- Was the scientific quality of the included studies assessed? (e.g. Cochrane risk of bias, Jadad scale, etc.)

Randomized controlled trials were appraised with a modified Cochrane Risk of Bias framework with a focus on two domains, plus the source of funding:

- Risk of bias arising from the randomization process.
- Risk of bias due to deviations from the intended interventions.
- Reporting for the source of funding was also assessed.

The results from the literature searches were compiled into evidence summaries for each of the 6 questions and presented to the Work Group for consideration through a modified Delphi process and via email. Disagreements were handled by email until consensus was reached.

### Research question 1

#### In patients with symptoms indicative of AR, is serum-specific IgE sufficient to identify candidates for immunotherapy or is a skin prick test mandatory?

A total of 45 articles were included after screening for the relevant inclusion and exclusion criteria for research question 1 (Table 1) [5, 11–54]. 

**Table 1:** Literature search results for research question 1

Clinical Practice Guidelines	Systematic Reviews	Randomized Controlled Trials	Comparisons of SPT and sIgE	SPT	sIgE Tests	Future Directions and Additional Studies of Interest
Dykewicz et al., 2020 [5]	Nevis et al., 2016 [35]	Visitsunthorn et al., 2017a [45]	Alimuddin et al., 2018 [13]	Al-Ahmad et al., 2021 [11]	Buzzulini et al., 2019 [16]	Arasi et al., 2021 [14]
Caro et al., 2016 [17]		Visitsunthorn et al., 2017b [46]	Bignardi et al., 2019 [15]	Al-Shagahin et al., 2019 [12]	Chang et al., 2021 [18]	Kim et al., 2019 [30]
Scadding et al., 2017 [40]		Visitunthorn et al., 2016 [47]	Chauveau et al., 2017 [19]	Corsico et al., 2017 [21]	Chen et al., 2017 [20]	Letrán et al., 2021 [31]
Wise et al., 2018 [50]			Gogunskaya et al., 2020 [23]	González-Pérez et al., 2020 [25]	Di Fraia et al., 2019 [22]	Pang et al., 2021 [36]
			González-Mancebo et al., 2017 [24]	Hurst and McDaniel, 2021 [27]	Kim et al., 2021 [29]	Stemeseder et al., 2018 [43]
			Hong et al., 2018 [26]	Ibekwe and Ibekwe, 2016 [28]	Park et al., 2018 [37]	Xie et al., 2021 [51]
			Nam et al., 2021 [34]	Shyna et al., 2018 [41]	Yang et al., 2018 [52]	
			Sağlam et al., 2016 [38]	Madani et al., 2021 [32]		
			Saltabayeva et al., 2017 [39]	Mostafa et al., 2019 [33]		
			Srisuwatchari et al., 2020 [42]	Wang et al., 2017 [48]		
			Traiyan et al., 2021 [44]			
			Wanjun et al., 2018 [49]			
			Zidarn et al., 2019 [53]			
			Zubchenko et al., 2019 [54]			

sIgE, serum-specific immunoglobulin E; SPT, skin prick test

### Evidence-based response

Allergic rhinitis is considered to be an immunoglobulin E (IgE)-mediated process [50], and a test of the allergic process and the specific allergens that trigger it is suggested by several guidelines to differentiate AR from other forms of rhinitis. If not performed earlier in the clinical workup, a test of the IgE presence to a specific allergen is required to prescribe and guide AIT.

Two common forms of testing for the presence of IgE are a skin-prick test (SPT) and a blood-test-based serum-specific IgE (sIgE) test. All clinical practice guidelines reviewed here include both SPT and serum IgE as acceptable options for identifying IgE-mediated reactions [5, 17, 40, 50], e.g.:

- The Rhinitis Practice Parameter states “AIT should be considered for patients with AR who have specific IgE antibodies to clinically relevant allergens…” “We recommend that aeroallergen skin prick testing **or** sIgE testing be completed to confirm the diagnosis of AR in a patient with a history consistent with AR.” [5] (emphasis added).
- The Philippine Society of Otolaryngology – Head and Neck Surgery guidelines state: “Detailed allergic work-up, e.g., skin tests, serum specific IgE tests, or nasal provocation tests, may be performed for the following: […] Patients for whom immunotherapy is considered[…]” “Specific IgE testing is indicated to provide evidence of an allergic basis for the patient’s symptoms, to confirm or exclude suspected causes of the patient’s symptoms, or to assess sensitivity to specific allergens for avoidance measures and/or allergen immunotherapy.” “In general practice, if skin tests are not readily available, serum specific IgE tests may be carried out.” [17].
- The British Society of Allergy an Clinical Immunology (BSACI) guidelines state: “Allergen-specific IgE can be detected with SPTs or by serum immunoassay. Skin prick tests should be carried out routinely to determine if the rhinitis is allergic or non-allergic…” “Serum-specific IgE may be requested when skin tests are not possible…” “Allergen immunotherapy within the United Kingdom is recommended in patients with a history of symptoms on allergen exposure and objective confirmation of IgE sensitivity (skin prick test positive and/or elevated allergen-specific IgE)…” [40].
- The International Consensus statement on Allergy and Rhinology: Allergic Rhinitis (ICAR: AR) states: “…a SPT or in vitro antigen-specific IgE (sIgE) test can be used to confirm the diagnosis of AR.” In sections dedicated to each test, Wise et al. [50] confirm the value of both SPT and sIgE for guiding immunotherapy. “Skin testing is crucial to directing AIT, and therefore, should be utilized in eligible patients when AIT is being considered.” “…sIgE may help in the selection of candidates for AIT and possibly predict the response.” [50].

Various trade-offs exist for the choice of SPT and sIgE. The SPT is universally accepted as the diagnostic method of choice for environmental allergy with sIgE generally provided as an alternative (rather than first) choice. However, SPT requires more skill on the part of the practitioner and carries certain risks for the patient (adverse events including discomfort and local allergic reactions; very low risk of anaphylaxis) and inconveniences (halting the use of medications that may confound the skin response). Other factors that influence the accuracy of SPT and make it difficult to compare studies include the stability and potency of the testing reagents, reactivity on the day of testing, variability by testing site, and skin color [35]. 

In comparison, sIgE is a safe and effective alternative, can permit the testing/screening of a wider panel of allergens, and the results are generally less variable [11–13]. 

The guidelines, reviews, and research studies could not have foreseen the impact of COVID-19 on the care and management of AR, however the context of the COVID-19 pandemic should qualify as an acceptable reason for choosing sIgE testing over SPT to guide the use of immunotherapy in AR. Indeed, in 2016, the Philippine Society of Otolaryngology speculated that advancing technology and the standardization and ability to use larger panels of allergens in tests would likely lead to sIgE supplanting SPT in the future [17]. 

In support of the use of sIgE for guiding immunotherapy, ICAR: AR [50] suggest that sIgE levels may correlate better with those patients more likely to benefit from AIT. And all sources suggest that, in general, SPT and sIgE correlate well for diagnosing AR. E.g., “There is good evidence to show that sIgE is, in many ways, equivalent to SPT,” summarizes ICAR: AR [50] Saltabayeva et al.’s [39] study similarly found that sIgE was more precise than SPT in identifying genuine sensitizations as targets for AIT, and that sIgE testing identified fewer patients as cross-reacting to multiple types of pollen than SPT, a finding that would save costs on AIT well beyond the cost differential of sIgE over SPT. Beyond the initial selection for immunotherapy, changes in sIgE levels following therapy can predict patient satisfaction with immunotherapy [29]. 

The strength of that conclusion depends on whether cost and speed of obtaining results are factors in the clinical decision, and on the relative sensitivity and specificity of the approaches. Here the literature is not conclusive, though sIgE testing demonstrates sensitivities of 67–96% and specificities of 80–100% (summary from ICAR: AR [50]). Traiyan et al. [44] found similarly high sensitivities and sensitivities for SPT (90% sensitivity and 88.3% specificity) and sIgE (89% and 95%, respectively).

The performance may vary for specific allergens. In the BSACI guideline [40] summary of the literature, SPT and sIgE had similar sensitivities for dust mites, but SPT was more sensitive for cat (also in Nam et al [34]), mold, and pollen allergens. Bignardi et al. [15] found a similar ranking, though all allergens tested had AUCs of over 84% and only the sensitivities of dog and tree pollen by sIgE versus SPT were under 80%. Conversely, Visitsunthorn et al. [45] found excellent agreement between SPT and sIgE for cat (and dust mite) allergens. Comparing SPT and sIgE for dust mites, Alimuddin et al. [13] found a high positive predictive value, but some patients with positive SPT had negative sIgE results (sensitivity below 50% for one particular dust mite and a cockroach allergen). Chauveau et al. [19] found only moderate agreement between SPT and sIgE, but that both had similar predictive ability for allergic diseases, reinforcing ICAR: AR’s [50] point that the choice of gold standard can influence the comparison. Hong et al. [26] suggest adjusting the cutoff value of sIgE tests for each allergen, though this approach may require additional study before clinical implementation.

In conclusion, evidence suggests that either sIgE testing or SPT can be used to support the final diagnosis of AR and guide AIT. While sIgE testing was used much more routinely than SPT in the context of the COVID-19 pandemic, sIgE should continue to be accepted as a sufficient approach for guiding the use of immunotherapy in AR.

### Considerations and limitations regarding the body of literature

There are some limitations or other notable features regarding the body of evidence on this topic. Though some clinical practice guidelines, systematic reviews, and randomized controlled trials were identified through this search (Table 1), much of the literature identified was lower in the hierarchy of quality of evidence. This included original research such as cohort studies, retrospective studies, comparative studies, cross sectional studies, and observational studies.

It is also important to note the variations in populations and allergens included across studies. While all the included studies included participants with allergic rhinitis, some studies also included additional participants with other respiratory or allergic conditions such as asthma or food allergies. Additionally, some studies may not be as applicable to the Canadian context given the specific allergens considered (e.g., cockroach, local flora) [13, 42, 43]. The heterogeneous nature of these studies makes it challenging to synthesize the evidence in this area.

Many of the articles identified in this search focused on comparing diagnosis of allergic rhinitis SPT and serum sIgE (Table 1). These studies may utilize different allergens but often focus on identifying the sensitivity and specificity of the tests when diagnosing allergic rhinitis and identifying specific allergens to which patients are allergic or sensitive.

Some included studies compared either the SPT or serum sIgE test to other allergy diagnostic tests or examined the value and nuances of the tests in question themselves. While these studies do not provide direct comparisons between the two tests, they may provide relevant information and a deeper understanding of SPT and serum sIgE testing. Another consideration is the differences in responses to the commercially available extracts in Canada [55]. Some studies found in the search offer a look at other types of component resolved diagnostics or other avenues of diagnosis that may not be common practice in Canada, but may still be of interest to this research question.

### Research question 2

#### When taking into account the preferences of the patient and the prescriber (stakeholder engagement) should second generation oral antihistamine or intranasal corticosteroid be first line?

A total of 39 articles were included after screening for the relevant inclusion and exclusion criteria for research question 2 (Table 2) [4, 5, 40, 50, 56–90]. 

**Table 2:** Literature search results for research question 2

	Systematic Reviews			Randomized Controlled Trials		
Clinical Practice Guidelines	OAH & INCS	OAH	INCS	OAH & INCS	OAH	INCS
Brożek et al., 2017 [56]	Juel-Berg et al., 2017 [62]	Huang et al., 2019 [61]	Donaldson et al., 2020 [57]	Wartna et al., 2017 [82]	Hashiguchi et al., 2017 [59]	Ellis et al., 2016 [58]
Dykewicz et al., 2017 [4]	Meltzer et al., 2021 [67]	Liu et al., 2018 [65]	Hoang et al., 2022 [60]		Locks et al., 2017 [66]	Karaulov et al., 2019 [63]
Dykewicz et al., 2020 [5]	Zhang et al., 2022 [88]	Miligkos et al., 2021 [68]	Khattiyawittayakun et al., 2019 [64]		Nayak et al., 2017 [69]	Ng et al., 2021 [70]
Scadding et al., 2017 [40]		Singh Randhawa et al., 2021 [77]	Phinyo et al., 2022 [76]		Nourollahian et al., 2020 [71]	Noyama et al., 2016 [73]
Wise et al., 2018 [50]		Tiamkao et al., 2021 [79]	Valenzuela et al., 2019 [80]		Novak et al., 2016 [72]	Thongngarm et al., 2021 [78]
		Velentza et al., 2020 [81]	Wu et al., 2019 [84]		Okubo et al., 2019 [75]	Zieglmayer et al., 2020 [90]
		Wei, 2016 [83]			Okubo et al., 2017 [74]	Zhang et al., 2021 [89]
		Xiao et al., 2016 [85]			Yamprasert et al., 2020 [86]	
					Yonekura et al., 2019 [87]	

INCS, intranasal corticosteroid; OAH, oral antihistamine

### Evidence-based response

Second-generation OAH and intranasal corticosteroids (INCS) have evidence suggesting that they are safe and effective choices for AR and could be options for first-line therapies. Combination therapy has also been investigated, with a combination nose spray available on the market.

The identified guidelines ultimately recommend both intranasal antihistamines (INAH) and INCS as options for first-line therapies, with slightly stronger language around the choice of INCS as first-line therapy [4, 5, 40, 50, 56]. More severe cases of AR lead to stronger recommendations for INCS or combinations of INAH/INCS, while mild-to-moderate recommendations are more open toward INAH/INCS in combination. From the guidelines:

- The Allergic Rhinitis and its Impact on Asthma (ARIA) guideline states: “In patients with SAR, we suggest either a combination of an INCS with an OAH or an INCS alone… In patients with perennial AR (PAR), we suggest an INCS alone rather than a combination of an INCS with an OAH…” “We suggest a combination of an INCS/INAH rather than an INAH alone…” “In patients with SAR, we suggest an INCS rather than an INAH.” [56].

The ARIA guideline acknowledges that the difference between an INCS and INAH is likely small, and that “This is a conditional recommendation, and thus different choices will be appropriate for different patients. **Clinicians must help each patient to arrive at a decision consistent with her or his values and preferences, considering local availability and costs**.” [56] (emphasis added).

- The Rhinitis Practice Parameter states “INCS remain the preferred monotherapy for persistent AR, but additional studies support the additive benefit of combination treatment with INAH/INCS…” [5] Yet they add that INAH and combination therapies be considered as options: “We recommend that the clinician offer INAH as an initial treatment **option** for patients with SAR…” [5] (emphasis added) “We suggest that the clinician consider the combination INAH/INCS for moderate/severe SAR and PAR that is resistant to pharmacologic monotherapy.” [5] However, for *intermittent* AR, their treatment pathway suggests INAH as the first treatment option, likely because of the on-demand nature of intermittent AR. Having said that, INAH options in Canada are limited, so we have to defer to INAH/INCS options in this circumstance.
- The BSACI guidelines state: “[INCS are the] First-line therapy for moderate-to-severe persistent symptoms…” “[INAH are] …the first line of therapy for mild-to-moderate intermittent and mild persistent rhinitis.” “[INAH] are less effective than an INCS in relieving the symptoms of AR.” “There is a rapid onset of action (15 min), faster than OAH, thus, the drug can be used on demand as rescue therapy for symptom breakthrough. Continuous treatment is, however, more clinically effective than on demand use.” [40] Again, having said that, INAH options in Canada are limited, so we have to defer to INAH/INCS options in this circumstance.
- ICAR: AR states: “INCSs are first-line therapy for the treatment of AR due to their superior efficacy in controlling nasal congestion and other symptoms of this inflammatory condition. Subjects with known SAR should start prophylactic treatment with INCS several days before the pollen season… The well-proven efficacy of INCSs, as well as their superiority over other agents, make them first-line therapy in the treatment of AR.” “INAH may be used as first-line or second-line therapy in the treatment of AR.” [50].

Patient preferences and co-morbidities certainly play a role in influencing the choice of treatment; the ARIA guideline suggests that the choice would depend mostly on patient preferences and other factors [56]. 

When making a patient-specific decision, some factors to consider include the potential side effects, the symptoms to be controlled and the severity of the AR, and any co-morbidities the patient may have, as well as the patient and provider preferences, the cost and availability of the medications, and any issues regarding treatment compliance.

In particular, INAH may have a bitter taste, while INCS may cause irritation, dryness and epistaxis. There are some reports of increased intra-ocular pressure with INCS, although studies did not show a significant increase in intra-ocular pressure when given over one year of therapy [91–93]. There are also concerns that some INCS may affect growth in children [50]. Long-term once-daily mometasone and fluticasone propionate in children given at the recommended dosage and once-daily budesonide at a dose of 64 mcg have not demonstrated any growth suppression [94–96]. INCS may also have sensory attributes that can affect patient preference and adherence to therapy, including aftertaste, nose runout, throat rundown, and smell [5]. 

In terms of symptom control, INAH are more effective for ocular symptoms and sneezing than INCS [50], though when used on as-needed basis, Hoang et al. [60] found on-demand INCS superior to OAH for sneezing, though INCS will control nasal symptoms better, particularly nasal congestion. The guidelines agree that INCS should be the first-line therapy for patients with more severe symptoms. For intermittent AR or for as-needed use, INAH have a faster onset [40, 50]. 

A full consideration of co-morbidities was not examined for this summary, however some main points that stood out in the literature include the potential for increased intra-ocular pressure with INCS, suggesting that they may need to be used with caution in patients at risk for glaucoma [40]. INCS may have benefits for other conditions, in particular ICAR: AR [50] suggest that INCSs may improve asthma control measures in patients suffering from both AR and asthma.

Patient compliance and preferences may change the recommendations for treatment, however. INCS are generally recommended for daily use, with the effect building over the course of approximately 2 weeks; for SAR the suggestion is to begin prophylactic treatment in advance of allergen exposure [50]. However, several studies raised the concern that patients use medication on demand, and stop when symptoms are controlled [60, 70]. Phinyo et al. [76] cite evidence that “the vast majority of AR patients are not adherent to their medication.” Ng et al. [70] characterize it:

“…the sensation of sprayed liquid in the nose may lead patients to mistakenly believe that intranasal steroid sprays work instantly. […] patients looking for rapid relief from allergy symptoms and congestion may self-treat with an INCS, unaware of their prolonged onset of action relative to oral agents. The locally applied nasal spray often confuses unaware patients (based on clinical practice experience, internal market research, and online consumer product reviews), who do not realize that the local spray sensation is not indicative of a symptom relief. Though the sensation in the nose may last for a short period of time, the patient may discontinue the use of INCS after administering the first dose, believing the treatment is not working.” [70].

Several studies have looked at the use of INCS in as-needed form vs. regular dosing, and universally found symptom relief, in some cases statistically similar to regular use [60, 76, 78, 82]. However, Hoang et al. [60] suggest that physicians communicate with patients about medication use and emphasize the importance of adherence – the regular use of INCS prevents inflammation from recurring. Their meta-analysis found that regular use of INCS led to greater improvements in symptom measures than as-needed INCS, yet even that was superior to as-needed antihistamines.

Beyond the issue of regular INCS compliance, patient preferences may come into play with the use of any nasal spray (INCS or INAH/INCS) over oral medications. The guidelines generally agree that INAH are more effective choices than OAH, with a faster onset and improved relief of nasal congestion. The Rhinitis Practice Parameter [5] puts it most directly: “INAH are equal to or superior to OAH.” However, both INCS and INAH may have sensory side effects (e.g. bad taste) that some patients may not want.

Oral antihistamines remain an alternative treatment that has been well-studied. Second-generation OAH provide effective symptom control with generally acceptable safety profiles and side effects and are widely available as over-the-counter and prescription-only medications. While the time to onset of symptom relief is slower than INAH, OAH can have faster onsets than INCS [40, 50]. Though not recommended in the examined guidelines as first-line therapies other than for mild intermittent symptoms, there is a place for them in the treatment options of each guideline reviewed.

In conclusion, existing guidelines generally agree on the use of INCS as a first-line therapy used for AR, however, patient and provider preferences and considerations can easily shift the first choice to a second-generation OAH. Second-generation OAH are widely supported as treatment options, with evidence of their efficacy in symptom control.

### Considerations and limitations regarding the body of literature

While all of the included studies considered subjects with AR, a few also included additional participants with other related respiratory or allergic conditions such as asthma. While some literature was identified on the direct comparison of second-generation OAH and INCS, a great deal of the included literature examined each of these treatments in comparison to other treatment options, such as combination therapy or leukotriene receptor antagonists. Others examined different options within OAH or INCS themselves. While allergens relevant to AR were considered across the different studies, the researchers may have included allergens that are more prevalent in other geographic locations but not in Canada. Additionally, a wide variety of possible treatments were included as comparisons to OAH and INCS.

### Research question 3

#### Is a combination INAH/INCS formulation superior to INCS plus OAH? Do they become equivalent after prolonged use?

A total of 30 articles were included after screening for the relevant inclusion and exclusion criteria for research question 3 (Table 3) [4, 5, 40, 50, 56, 97–121]. 

**Table 3:** Literature search results for research question 3

Clinical Practice Guidelines	Systematic Reviews	Randomized Controlled Trials	Other Original Studies of Interest
Brożek et al., 2017 [56]	Abd El-Raouf et al., 2020 [97]	Andrews et al., 2020 [99]	Agache et al., 2018 [98]
Dykewicz et al., 2017 [4]	Chen et al., 2022 [102]	Bousquet et al., 2018 [100]	Canonica et al., 2021 [101]
Dykewicz et al., 2020 [5]	Chitsuthipakorn et al., 2022 [103]	Gross et al., 2019 [107]	Haahr et al., 2019 [108]
Scadding et al., 2017 [40]	Debbaneh et al., 2019 [104]	Hampel et al., 2019 [109]	Kaulsay et al., 2018 [110]
Wise et al., 2018 [50]	Du et al., 2020 [105]	Kortekaas Krohn et al., 2018 [114]	Klimek et al., 2016 [111]
	Feng et al., 2016 [106]	Patel et al., 2019 [115]	Klimek et al., 2017 [112]
	Seresirikachorn et al., 2018 [118]	Segall et al., 2019 [117]	Klimek et al., 2020 [113]
	Zhong et al., 2022 [121]		Scadding et al., 2017 [116]
			Stjarne et al., 2019 [119]
			Watts et al., 2022 [120]

### Evidence-based response

The evidence consistently suggests that the combination of an INAH/INCS is superior to an INCS plus OAH. Indeed, many studies find that there is no benefit from adding an OAH to an INCS over the symptom relief provided by the INCS alone.

- The Rhinitis Practice Parameter [5] suggests the combination of INAH/INCS in certain circumstances (as first-line for patients with moderate/severe AR, or for those who are resistant to monotherapy), or OAH alone, but not the combination of OAH and INCS: “We suggest that the clinician not prescribe the combination of an OAH and an INCS in preference to monotherapy with an INCS in all patients with SAR and PAR.”
- ICAR: AR states: “Combination therapy of INCS and OAH does not improve symptoms of nasal congestion over INCS use alone, and does risk the adverse effects of systemic antihistamine use.” “Combination therapy with INAH/INCS may be used as second-line therapy in the treatment of AR when initial monotherapy with either INCS or antihistamine does not provide adequate control.” [50].
- Abd El-Raouf et al. state: “In this study have we aimed to determine whether the additional effects of INAH to INCS may play a role in rapid improvement of allergic symptoms during the 2-week period needed for the INCS to achieve maximum effect. Our findings show greater benefits with use of INAH/INCS over INCS alone. The effects were significant only for the INAH/INCS combination. […] Based on our findings, the combination of INCS and OAH is not recommended…” [97].
- Chitsuthipakorn et al. state: “The pooled data showed that the effects of the INAH/INCS combination were better than INCS alone on the improvements of composite nasal symptom score […] The INCS-OAH combination did not show any beneficial effects.” [103].
- Du et al. state: “INAH have an add-on effect on INCS, and the combination of INAH/INCS is superior to that of OAH plus INCS in improving nasal symptoms for patients with AR.” [105].

Similarly, Feng et al.’s [106] review found that INCS plus OAH had similar efficacy to INCS alone, whereas a meta-analysis of 6 randomized controlled trials did demonstrate that the combination of INAH/INCS was superior to INCS alone (and thus, presumably also to INCS plus OAH) [118].

- Seresirikachorn et al. state: “Subgroup analysis indicated no benefit with the OAH-INCS combination but did show benefit with INAH/INCS.” [118].

However, there is insufficient evidence to answer the second question of whether a combination INAH/INCS formulation becomes equivalent to INCS plus OAH after prolonged use. There were no long-term head-to-head studies within the parameters of the literature search, indeed there are very few long-term studies of combination therapies at all.

- Abd El-Raouf et al. state: “… all 4 studies assessing this combination only analyzed data at the single time-point of 2 weeks. It is not known whether these additional effects persist after a 2-week duration.” [97].
- Chitsuthipakorn et al.’s [103] analysis included one study that ran to 90 days of usage, which indicated that the effects of INAH/INCS continued to be sustained at least for that length of time.

In one study that did include up to 52 weeks of data, the effectiveness of INAH/INCS in AR symptom control was compared to placebo, and they found differing results for the 52-week time period depending on the measure, with reflective Total Nasal Symptom Score and instantaneous Total Nasal Symptom Scores significantly improved vs. placebo (*p* < 0.001), whereas Physician-assessed Nasal Symptom Scores differences were not statistically significant from placebo at 52 weeks. [117] Segall et al. [117] concluded that the combination INAH/INCS treatment demonstrated long-term efficacy. However, in their meta-analysis that included that study, Chen et al. [102] conclude that INAH/INCS “…can only relieve the nasal and eye symptoms of AR patients in the short term, but cannot control rhinitis symptoms and improve the quality of life in the long term.” However one is to interpret that study, it only looked at INAH/INCS vs. placebo, and there remains a knowledge gap about the choice of INCS plus OAH or either individually vs. INAH/INCS over the long term.

An observational survey-based study does support the long-term use of INAH/INCS [101]. Participants had begun combination therapy an average of 2.6 years prior to the survey, and 70% reported that they were more or much more satisfied with it than their previous AR treatment (which was not specified). “Significantly (*p* < .001) more participants (76%) were more or much more satisfied with Meda Pharmaceutical azelastine fluticasone propionate (MP-AzeFlu) than their previous AR treatment when MP-AzeFlu was used every day symptoms were expected, compared to 66% of participants who used it only on symptom days,” [101] which speaks to the importance of treatment compliance in long-term patient satisfaction.

In conclusion, the combination INAH/INCS is superior to an INCS plus OAH. However, there was insufficient evidence to answer the second question of whether they become equivalent after prolonged use.

### Research question 4

#### Do leukotriene receptor antagonists (LTRA) have a greater benefit than OAH in allergic rhinitis for some symptoms to justify a therapeutic trial in those who cannot tolerate INCS?

A total of 26 articles were included after screening for the relevant inclusion and exclusion criteria for research question 4 (Table 4) [4, 5, 40, 50, 56, 81, 83, 85, 122–139]. 

**Table 4:** Literature search results for research question 4

Clinical Practice Guidelines	Systematic Reviews	Randomized Controlled Trials	Other Original Studies of Interest
Brożek et al., 2017 [56]	Feng et al., 2021 [127]	Kim et al., 2018 [130]	Andhale et al., 2016 [122]
Dykewicz et al., 2017 [4]	Krishnamoorthy et al., 2020 [131]	Okubo et al., 2017 [133]	Bhattachan et al., 2020 [123]
Dykewicz et al., 2020 [5]	Velentza et al., 2020 [81]		Bian et al., 2021 [124]
Scadding et al., 2017 [40]	Wei, 2016 [83]		Dalgic et al., 2017 [125]
Wise et al., 2018 [50]	Xiao et al., 2016 [85]		Durham et al., 2016 [126]
			Jindal et al., 2016 [128]
			Kaur et al., 2017 [129]
			Li et al., 2018 [132]
			Sansing-Foster et al., 2021 [135]
			Rajput et al., 2020 [134]
			Whalley et al., 2017 [136]
			Yoshihara et al., 2017 [137]
			Zhao et al., 2021 [138]
			Zuberi et al., 2020 [139]

### Evidence-based response

Several systematic reviews have examined the relative effectiveness of LTRAs (e.g., montelukast) and OAH, generally finding that LTRAs have inferior, or at best equivalent, daytime or overall symptom control [81, 83, 85, 127, 131]. 

However, studies suggest that LTRAs improve nighttime symptom control:

- Feng et al. state: “LTRAs are superior to OAHs for improving the nighttime symptoms of AR, including nasal congestion on awakening, difficulty going to sleep, and nighttime awakenings…” [127].
- Krishnamoorthy et al. state: “Although OAH was superior to montelukast in most of the relief of the symptoms in allergic rhinitis, our meta-analysis found montelukast was effective in improving [night-time nasal symptom score]. This suggests that montelukast may be used as an alternative to OAH as first-line therapy in allergic rhinitis especially for patients who have predominantly night-time symptoms.” [131].
- Wei et al. state: “Montelukast has a significant influence in improving patients’ nasal symptoms quality of live [sic] but is not as effective as OAHs, and may have a slight advantage over OAHs in relieving nighttime symptoms significantly.” [83].
- Velentza et al. state: “Interestingly, montelukast was shown to be more effective than cetirizine in improving night sleep quality, according to patients’ diaries.” [81].

LTRAs can also provide benefits in AR patients with asthma as a co-morbidity, which is common (40% per the Rhinitis Practice Parameter [5]).

- The Rhinitis Practice Parameter states “We recommend that the oral LTRA montelukast should only be used for AR in patients who have an inadequate response or intolerance to alternative therapies. […] In patients with AR comorbid with asthma, compared with placebo, montelukast could result in significant improvements in both conditions and therefore can be considered an option for patients with both conditions. However, due to the only modest efficacy and also the potential increased risks of montelukast compared with those of OAH, for the management of AR and comorbid asthma, the clinician should weigh the benefits of montelukast monotherapy versus an inhaled corticosteroid for asthma and an antihistamine or INCS for AR.” [5].
- The ARIA guideline states: “Some patients with AR who have concomitant asthma, especially exercise-induced and/or aspirin exacerbated respiratory disease, may benefit from LTRA more than from OAH[…] Patients with asthma who have concomitant AR should receive an appropriate treatment according to the guidelines for the treatment of asthma.” [56].
- The BSACI guidelines state: “[LTRAs] have a therapeutic profile similar to antihistamines, with efficacy comparable to loratadine in seasonal allergic rhinitis […] [LTRAs] may have a place in asthma patients with SAR.” [40].
- ICAR: AR states: “In patients with concurrent AR and asthma, LTRA can contribute to symptom management of both respiratory diseases. LTRA monotherapy is not recommended as first-line treatment for patients with concurrent AR and asthma, although this may be a consideration in patients with contraindications to INCS.” [50].

Thus, there may be a justification to conduct a therapeutic trial if either the control of nighttime symptoms is a priority, or in cases of AR with asthma. However, few studies directly compared LTRAs to OAHs, and most randomized controlled trials did not include both an OAH and LTRA treatment arm.

Kim et al. [130] studied people with AR and mild-to-moderate asthma and found a greater benefit of combined LTRA plus OAH therapy to LTRA alone. However, they did not examine LTRA vs. OAH alone, nor other treatment combinations in those with asthma (e.g., LTRA for controlling both upper and lower airway symptoms vs. separate inhaled corticosteroids for lower airway symptoms and OAH/INAH/INCS for upper airway/AR symptoms).

A small study of children in Japan with asthma and AR (20 patients per group) found that AR symptoms were better controlled through the beginning of the pollen season when LTRA was given continuously, versus those who were given either LTRA or OAH on demand (waiting for symptoms to start after pollen season began) [137]. This difference in approach for those with SAR and asthma may be of interest for those who cannot tolerate other treatments.

A note of caution, however, the Rhinitis Practice Parameter [5] and Bian et al. [124] report that LTRAs can have neuropsychological side effects, particularly in children under 6. However, in limiting their data to patients over 6 with asthma, Sansing-Foster et al. [135] did not find an increased risk of hospitalizations for depression or self-harm and found that 93% of patients with a psychiatric adverse event were those with a history of psychiatric disorder.

In conclusion, LTRAs have inferior, or at best equivalent, daytime or overall symptom control compared with OAH. However, LTRAs may improve nighttime symptom control and provide benefits in patients with AR and concomitant asthma.

Considerations and Limitations Regarding the Body of Literature.

Studies comparing the effectiveness of LTRAs with OAH have been limited to SAR and are lacking for PAR.

### Research question 5

#### Should sublingual immunotherapy (SLIT) tablets be considered first-line immunotherapeutic options over subcutaneous immunotherapy (SCIT) based on the evidence of efficacy?

A total of 85 articles were included after screening for the relevant inclusion and exclusion criteria for research question 5 (Table 5) [5, 40, 50, 67, 126, 140–219]. 

**Table 5:** Literature search results for research question 5

	Systematic Reviews			Randomized Controlled Trials			
Clinical Practice Guidelines	Comparison	SCIT	SLIT	Comparison	SCIT	SLIT	Other Studies Focused on Long-Term Treatment
Dykewicz et al., 2020 [5]	Elliott et al., 2017 [141]	Kim et al., 2021b [142]	Boldovjáková et al., 2021 [145]	Shamji et al., 2021 [157]	Bozek et al., 2016 [151]	Barker-Tejeda et al., 2021 [162]	Albuhairi et al., 2018 [186]
Roberts et al., 2018 [140]	Kim et al., 2021a [143]		Chen et al., 2020 [146]	Xian et al., 2020 [161]	Bozek et al., 2017 [150]	Bernstein et al., 2018 [163]	Antico 2022 [187]
Scadding et al., 2017 [40]	Tie et al., 2022 [144]		Feng et al., 2017a [147]		Chaker et al., 2016 [152]	Biedermann et al., 2019 [164]	Asaumi et al., 2021 [188]
Wise et al., 2018 [50]			Feng et al., 2017b [148]		Pfaar et al., 2017 [154]	Birk et al., 2017 [165]	Baba et al., 2021 [189]
			Li et al., 2018 [149]		Pfaar et al., 2016 [155]	Couroux et al., 2019 [166]	Borg et al., 2020 [190]
			Meltzer et al., 2021 [67]		Scadding et al., 2017 [156]	Demoly et al., 2021 [167]	Chen et al., 2019 [191]
					Sola et al., 2016 [158]	Demoly et al., 2016 [168]	Devillier et al., 2017 [192]
					Worm et al., 2018 [159]	Ellis et al., 2018 [169]	Di Bona et al., 2020 [193]
					Worm et al., 2019 [160]	Emminger et al., 2017 [170]	Droessaert et al., 2016 [194]
						Gotoh et al., 2019 [171]	Durham et al., 2016 [126]
						Ihara et al., 2018 [172]	Fan et al., 2016 [195]
						Jerzynska et al., 2016 [173]	Fritzsching et al., 2022 [196]
						Mäkelä et al., 2018 [174]	Fujisawa et al., 2018 [197]
						Maloney et al., 2016 [175]	Gelincik et al., 2017 [198]
						Masuyama et al., 2018 [153]	Halken et al., 2020 [199]
						Mösges et al., 2017 [176]	Huang et al., 2019 [200]
						Nolte et al., 2020 [177]	Huoman et al., 2019 [201]
						Nolte et al., 2016 [178]	Kiotseridis et al., 2018 [202]
						Nolte et al., 2021 [179]	Lim et al., 2017 [203]
						Okamoto et al., 2017 [180]	Lourenco et al., 2020 [204]
						Okubo et al., 2017 [181]	Malet et al., 2016 [205]
						Roux et al., 2016 [182]	Manzotti et al., 2016 [206]
						Valovirta et al., 2018 [183]	Otsuka et al., 2020 [207]
						Yonekura et al., 2019 [184]	Pavon-Ramero et al., 2021 [208]
						Yonekura et al., 2021 [185]	Pawlowski et al., 2017 [209]
							Pothirat et al., 2021 [210]
							Sahin et al., 2017 [211]
							Schmid et al., 2021 [212]
							Ünal, 2020 [213]
							Varona et al., 2019 [214]
							Vogelberg et al., 2020 [215]
							Wahn et al., 2019 [216]
							Yang et al., 2021 [217]
							Zhu et al., 2021 [218]
							Zielen et al., 2018 [219]

SCIT, subcutaneous immunotherapy; SLIT, sublingual immunotherapy

### Evidence-based response

Recent clinical practice guidelines discuss the trade-offs involved with AIT, including SLIT and SCIT [5, 40, 50, 140]. These include the potential side effects (both), the need to identify the particular allergen(s) causing AR symptoms (both), the need for office visits (SCIT), provider expertise for preparation and delivery (SCIT), cost (both), and sustained compliance to modify the immune response (both). However, neither has a strong recommendation over the other on the basis of efficacy.

Randomized controlled trials and systematic reviews published since 2016 examining comparative efficacy after long-term therapy provide mixed evidence depending on the allergen examined and outcomes considered.

- Elliott et al. [141] conducted a review of both types of AIT and found limited data for the comparison. SCIT out-performed SLIT in improving medication and symptom scores, though both treatment approaches were equivalent for improving quality of life scores. “The findings of our review suggest that both SCIT and SLIT are effective compared with placebo, but further work may be required to establish their comparative efficacy.”
- Kim et al. [143] compared studies of SCIT and SLIT for dust mite allergies and suggested that SCIT may be more effective, though only 1 of the SCIT studies included in their meta-analysis had a treatment duration of 3 years.
- Tie et al. [144] found no significant differences between SCIT and SLIT in their indirect comparison meta-analysis. Only 2 of their included studies used treatments of 3 years. “Based on results from an adjusted indirect comparison, treatment with either SCIT or SLIT results in comparable patient outcomes, so the decision for choosing between the two may be guided by other considerations, such as availability, cost, and patient preference.”
- Shamji et al. [157] reported that both treatments improved nasal symptoms after two years of treatment for grass pollen, but neither maintained that improvement after a year off treatment.
- Xian et al. [161] compared SCIT and SLIT in house dust mite allergies and found both were effective vs. placebo, with no differences across most measures, with SCIT having a slightly better (statistically significant; *p* = 0.026) improvement on the medication score, though in their own words “But it was not enough to state that SCIT works better than SLIT.”
- Using a 2-year dosing regimen and 1-year follow-up period, Scadding et al. [156]. found that SCIT was effective, while SLIT was not. This may be taken as evidence that SCIT is more robust to changes in treatment duration than SLIT, though it perhaps underscores the need for full treatment compliance in AIT (especially SLIT) rather than suggesting that SCIT is more effective than SLIT in 3-year regimens.
- Conversely, Gotoh et al. [171] found that a partial SLIT treatment period from their cross-over design was effective. They address the difference between their results and Scadding et al’s [156], suggesting that the different assessment methods may be a factor (allergen challenge vs. natural exposure). The different allergens used (grass vs. Japanese cedar) may also be a factor. However, Gotoh et al. [171] did not include a SCIT arm, limiting the applicability of this evidence for the core question.

Due to the dearth of recent, high-quality studies examining long-term therapy, the search strategy was expanded to include lower quality studies, which reported promising results of SCIT or SLIT treatment after 3 years.

- In an observational study, Droessaert et al. [194] reported that 70% of patients receiving SCIT for AR were not using any medication for their AR 3 years after starting their treatment, and the immunotherapy group had reduced symptom severity than those taking medications.
- In an unblinded study that did not report on the randomization process, Ünal [213] found a greater improvement in nasal symptoms (Total Rhinitis Symptom Score) from dust mite and *Parietaria* SCIT 3 years after starting treatment with SCIT than from pharmacotherapy.
- Baba et al. [189] examined rhinitis patients treated with SLIT vs. pharmacotherapy over 3 years and found sustained clinical improvement and a reduction in medication use from SLIT.
- Devillier et al. [192] conducted an observational study of grass SLIT tablets, including patients with up to 6 years of follow-up, and found reduced AR medication prescription and reduced risk of developing asthma (OR = 0.72). SLIT was administered to patients with AR for ≥ 3 years in 59% and 70% of patients receiving 5- and 1-grass-pollen SLIT tablets, respectively.
- In the REACT study, a retrospective cohort study of patients using AIT for at least one year, Fritzsching et al. [196] found that the subjects treated with SCIT/SLIT had reduced AR prescriptions compared with the control group which was sustained over 9 years.
- Huang et al. [200] studied adult and pediatric patients with AR who received house dust mite-SCIT for 3 years. After 3 years of treatment and 2 years following treatment, subjects showed improved symptoms and quality of life scores vs. pre-treatment, with children showing better improvements based on baseline compared to adults.
- Wahn et al. [216] studied a mix of SLIT and SCIT vs. pharmacotherapy in patients with birch pollen allergies who received treatment in ≥ 2 successive seasonal pollen cycles, with up to 6 years of follow-up data post-treatment. They found significantly more people in the AIT group were medication-free for AR and asthma symptoms (both *p* < 0.001), with many more reducing their medication use. The risk of developing new asthma was also reduced in the SCIT/SLIT treatment groups.

In general, both SCIT and SLIT have been shown to be effective for AR across a variety of allergens. However, without more direct long-term head-to-head studies or meta-analyses, it is difficult to draw conclusions regarding selecting one over the other on the basis of efficacy alone. Regardless, the differences in costs, risks, practical considerations for administration, and patient preferences may outweigh any quantifiable differences in efficacy [220–224]. 

In conclusion, the choice of SLIT or SCIT cannot be made on efficacy alone, and differences in other factors outweigh any differences in efficacy.

### Considerations and limitations regarding the body of literature

Allergen extracts used in SCIT and SLIT are highly variable in allergen content and potency, which can affect efficacy and safety. The use of extracts standardized for potency are necessary for high quality studies, and efficacy data associated with standardized extracts should not be extrapolated to non-standardized extracts. Furthermore, natural pollen exposures can also be a confounding variable for studies of SCIT/SLIT effectiveness, for example Worm et al. [160] found that SCIT was not more effective than placebo but noted that the pollen counts in the primary evaluation year were the lowest in the study period, so even the placebo group had reduced symptoms and medication use.

Limitations to the literature published since 2016 includes a focus on short-term treatments (most studies examined treatment of ≤ 2 years), and evaluation periods that coincided with the end of the treatment phase, rather than the following allergy season. Others examined various immunological biomarkers, rather than clinically relevant measures (e.g. Stylianou et al. [225]).

### Research question 6

### Based on efficacy data, should ALL patients seen by an allergist be offered SLIT or SCIT as a treatment option?

A total of 50 articles were included after screening for the relevant inclusion and exclusion criteria for research question 6 (Table 6) [5, 40, 50, 67, 140–185]. The search results were the same guidelines, systematic reviews, and randomized controlled trials identified for research question 5.

**Table 6:** Literature search results for research question 6

	Systematic Reviews			Randomized Controlled Trials		
Clinical Practice Guidelines	Comparison	SCIT	SLIT	Comparison	SCIT	SLIT
Dykewicz et al., 2020 [5]	Elliott et al., 2017 [141]	Kim et al., 2021b [142]	Boldovjáková et al., 2021 [145]	Shamji et al., 2021 [157]	Bozek et al., 2016 [151]	Barker-Tejeda et al., 2021 [162]
Roberts et al., 2018 [140]	Kim et al., 2021a [143]		Chen et al., 2020 [146]	Xian et al., 2020 [161]	Bozek et al., 2017 [150]	Bernstein et al., 2018 [163]
Scadding et al., 2017 [40]	Tie et al., 2022 [144]		Feng et al., 2017a [147]		Chaker et al., 2016 [152]	Biedermann et al., 2019 [164]
Wise et al., 2018 [50]			Feng et al., 2017b [148]		Pfaar et al., 2017 [154]	Birk et al., 2017 [165]
			Li et al., 2018 [149]		Pfaar et al., 2016 [155]	Couroux et al., 2019 [166]
			Meltzer et al., 2021 [67]		Scadding et al., 2017 [156]	Demoly et al., 2021 [167]
					Sola et al., 2016 [158]	Demoly et al., 2016 [168]
					Worm et al., 2018 [159]	Ellis et al., 2018 [169]
					Worm et al., 2019 [160]	Emminger et al., 2017 [170]
						Gotoh et al., 2019 [171]
						Ihara et al., 2018 [172]
						Jerzynska et al., 2016 [173]
						Mäkelä et al., 2018 [174]
						Maloney et al., 2016 [175]
						Masuyama et al., 2018 [153]
						Mösges et al., 2017 [176]
						Nolte et al., 2020 [177]
						Nolte et al., 2016 [178]
						Nolte et al., 2021 [179]
						Okamoto et al., 2017 [180]
						Okubo et al., 2017 [181]
						Roux et al., 2016 [182]
						Valovirta et al., 2018 [183]
						Yonekura et al., 2019 [184]
						Yonekura et al., 2021 [185]

SCIT, subcutaneous immunotherapy; SLIT, sublingual immunotherapy

### Evidence-based response

Allergen immunotherapy (both SLIT and SCIT) has been shown to provide clinical benefits in patients with AR and reduce or eliminate the need for pharmacotherapy. Overall effect shown varies depending on allergen being considered, length of treatment, and outcomes being measured.

Many studies have demonstrated the efficacy of SCIT and SLIT vs. placebo, some for a year or more after therapy ends [151, 156, 183, 185]. The results of studies are often more impressive than they first appear, as patients typically have access to pharmacotherapy (as rescue medications at least), so the SCIT/SLIT improvements are in addition to the benefits provided by symptom-controlling medications.

In addition, SLIT and SCIT have been shown to reduce the risk of a patient with AR later developing asthma [5, 40, 140, 183, 192, 216, 219]. The odds ratios were modest (e.g., the 0.66 in Valovirta et al. [183] was typical), however asthma is a common co-morbidity for AR so this could be clinically meaningful. Indeed, results from Valovirta et al. [183] suggest that the reduction in the risk and severity of asthma is itself a reason to use SLIT in patients with AR.

The efficacy for SLIT and SCIT suggests that these treatment approaches should be used broadly in patients with AR. However, that efficacy data must be balanced by other clinical concerns, which were not the target of this literature review, though some key themes are presented below.

Recent clinical practice guidelines discuss the trade-offs involved with AIT, including SLIT and SCIT [5, 40, 50, 140]. These include the potential side effects (both SLIT and SCIT), the need to identify the particular allergen(s) causing AR symptoms (both), the need for office visits (SCIT), provider expertise for preparation and delivery (SCIT), cost (both), and sustained compliance to modify the immune response (both). The guidelines generally agree that SLIT or SCIT be offered to patients whose AR is not well-controlled by pharmacotherapy or who have a preference for AIT.

Another concern is that AIT requires identifying the specific allergen(s) responsible for a patient’s symptoms and then using those allergens in the SLIT or SCIT. This testing burden may present a barrier to SCIT and SLIT treatment, as benefits of SLIT and SCIT are likely allergen specific (e.g. Ellis et al. [169] which showed no significant effect of Timothy grass SLIT in participants with birch pollen induced AR.)

Moreover, most patients exhibit polysensitization (many papers estimate the rate at 70–80% [226–228]), which confounds the choice and makes planning a course of treatment more difficult to implement. The European Academy of Allergy and Clinical Immunology guidelines for AIT on AR [140] suggest that AIT may be less effective or ineffective in polysensitized individuals, and if necessary, multiple allergens be given with a period of time between exposures, which adds to the implementation difficulties. ICAR: AR [50] suggest that in the US it is common to include multiple allergen extracts for SCIT, though this may not be possible for SLIT. They also suggest that more evidence is required to support the use of SCIT and SLIT in polysensitized individuals, although there is published evidence that AIT is effective in such patients [229–231]. 

In conclusion, the efficacy data suggests that SLIT or SCIT should be used broadly in patients with AR, but other clinical concerns also need to be taken into consideration.

### Considerations and limitations regarding the body of literature

Efficacy is only one aspect when considering the appropriateness of AIT for patients. The majority of patients will not need or want AIT for reasons of inconvenience, cost, or potential side effects, and the decision will be primarily up to patient preference [220–224, 232]. However, there are some patients in whom AIT may be more strongly recommended, such as asthmatics with clear worsening in response to allergic triggers or other comorbid atopic conditions.

## Discussion

Regional allergen sensitizations, medication product availability, healthcare systems, and other factors can influence the regional management of AR. Six questions specifically related to the clinical management of AR in Canada were identified by a Work Group of practicing Canadian physicians and recommendations were given based on reviews of the literature. The recommendations were not in the format of a standard guideline, but rather were focused as a series of Population, Intervention, Comparison, Outcome (PICO)-style questions which have become the norm in Traditional Practice Parameter statements from the Joint Task Force on Practice Parameters [233]. To this end, the Work Group determined there was sufficient evidence to conclude that: both sIgE testing and SPT are acceptable for diagnosing AR and guiding AIT; that INCS should generally be used as first-line therapy over OAH but is dependent on patient/provider preferences; that INAH/INCS is superior to an INCS plus OAH; that LTRAs are generally inferior, or at best equivalent, to OAH in controlling daytime symptoms but may improve nighttime symptom control and provide benefits with comorbid AR and asthma; that the choice between SLIT and SCIT cannot be based on efficacy alone; and that SLIT and SCIT should be broadly used in patients with AR but other clinical concerns also need to be taken into consideration.

Although several of the questions focused on decision-making based on efficacy, the recommendations for these questions tend to have caveats driven by patient preference. Does the patient prefer an OAH because the taste of INCS is intolerable? Is the patient willing to attend frequent office visits for SCIT? Such preferences can be determined in shared decision-making conversations that should be a part of each patient visit. Comorbidities, costs, and product availability are also considerations when choosing a treatment for AR. Thus, while the Work Group were able to make conclusions regarding efficacy based on published clinical guidelines and other literature sources, in many cases treatment decisions need to go beyond simple efficacy superiority.

A general limitation of this focused practice parameter is that pharmacoeconomics were considered, although cost and cost-effectiveness are certainly factors in treatment decisions. There is a large body of literature on pharmacoeconomics in AR that was beyond the scope for inclusion in this focused practice parameter.

## Conclusion

This focused practice parameter provides recommendations for specific questions in the management of AR in Canada. Implementation of these recommendations in conjunction with shared decision-making conversations may improve outcomes in Canadian patients with AR.

### Electronic supplementary material

Below is the link to the electronic supplementary material.

Supplementary Material 1

## Data Availability

Data sharing is not applicable to this article as no datasets were generated or analyzed during the current study.

## Abbreviations

AIT: Allergen immunotherapy
AR: Allergic rhinitis
AUC: Area under the curve
IgE: Immunoglobulin E
INAH: Intranasal antihistamines
INCS: Intranasal corticosteroids
LTRAs: Leukotriene receptor antagonists
OAH: Oral antihistamines
sIgE: Serum-specific IgE
SCIT: Subcutaneous immunotherapy
SLIT: Sublingual immunotherapy
SPT: Skin prick test
