# Supplementary material for: Focused allergic rhinitis practice parameter for Canada
Source: Allergy Asthma Clin Immunol. 2024 Aug 8;20:45. doi: 10.1186/s13223-024-00899-3 (PMC11311964; doi:10.1186/s13223-024-00899-3)
Supplement: Supplementary file 1 — Supplementary Material 1 [file 13223_2024_899_MOESM1_ESM.docx]

# Focused Allergic Rhinitis Guidelines for Canada

# Appendix 1

## Research Question 1: Is serum specific IgE sufficient to identify candidates for immunotherapy or is a skin prick test mandatory?

## Inclusion/Exclusion Criteria

A total of 868 results were returned in the database searches and 806 results were returned from searching the Internet and online resources. A reviewer screened these results for relevance to the research question and applied the inclusion and exclusion criteria for this project. Duplicate results from different searches were removed.

The inclusion criteria were as follows:

- Adult or pediatric participants with allergic rhinitis
- Focus on skin prick tests or serum specific IgE tests, ideally a comparison of the two tests
- Clinical practice guidelines, systematic reviews or meta-analyses, randomized controlled trials, or other original research, for example, cohort studies, comparative studies, cross sectional studies, retrospective studies, observational studies, clinical trials
- English language
- Published in the year 2016 or later
- Human subject studies

Exclusion criteria for the search was as follows:

- Articles focused on microarray technology or studies conducted with this technology without a comparison to skin prick testing or immunoassay sIgE testing, such as the ImmunoCAP, as it is not as relevant for a Canadian context. Component Resolved Diagnostic tests that were not focused on microarray tests were eligible for inclusion.
- Articles focused on RAST technology as this test is no longer clinically available
- Articles focused on the chemiluminescent method of immunoassay
- Articles focused on food allergy and not environmental allergens
- Articles focused on local allergic rhinitis, non-allergic rhinitis, occupational allergic rhinitis or other respiratory conditions such as asthma, that did not also include seasonal allergic rhinitis patients.

## Medline Search Strategy

Database: Ovid MEDLINE(R) ALL <1946 to March 18, 2022>

1 exp Rhinitis, Allergic/ (22761)

2 ((seasonal or intermittent or persistent or perennial or ragweed or grass or birch or house dust mite or mold or dander or cat or aeroallergen) adj4 (allergic rhinitis or allergic rhinitides)).mp. (3650)

3 1 or 2 (23444)

4 exp Immunoglobulin E/bl (15094)

5 exp Immunoglobulin E/im (14571)

6 (serum adj4 (IgE or immunoglobulin E) adj3 test*).mp. (987)

7 Immunocap.mp. (980)

8 sIgE.mp. (2148)

9 or/4-8 (28765)

10 skin prick test*.mp. (9052)

11 puncture test*.mp. (136)

12 scratch test*.mp. (1267)

13 or/10-12 (10445)

14 9 or 13 (36215)

15 3 and 14 (3622)

16 limit 15 to (english language and humans and yr="2016 -Current") (686)

## Cochrane Databases Search Strategy (March 22, 2022)

Databases: Cochrane Central Register of Controlled Trials, Cochrane Database of Systematic Reviews

Published Date: 2016/01/01-2021/12/31

S1 Allergic Rhinitis - Apply Equivalent subjects

S2 ((seasonal or intermittent or persistent or perennial or ragweed or grass or birch or house dust mites or mold or dander or cat or aeroallergen) n4(allergic rhinitis or allergic rhinitides)) - Apply Equivalent subjects

S3 S1 OR S2

S4 (serum n4 (IgE or immunoglobulin E) n3 test*) - Apply Equivalent subjects

S5 sIgE - Apply Equivalent subjects

S6 ImmunoCAP - Apply Equivalent subjects

S7 S4 OR S5 OR S6

S8 skin prick test* - Apply Equivalent subjects

S9 puncture test* - Apply Equivalent subjects

S10 scratch test* - Apply Equivalent subjects

S11 S8 OR S9 OR S10

S12 S7 OR S11

S13 S3 AND S12 (200)

## Internet Search Strategies

| Date | Repository | Search terms | # Results |
| --- | --- | --- | --- |
| March 17, 2022 | Google (first 5 pages) | - sIgE immunoassay allergic rhinitis - skin prick test allergic rhinitis | 50 |
| March 17, 2022 | CMA Infobase | - Allergic Rhinitis - Skin Prick Test - Puncture Test - Scratch Test - Serum IgE - IgE | 7 |
| March 17, 2022 | NICE | - Allergic Rhinitis - Serum IgE - IgE - Skin Prick Test - Scratch Test - Puncture Test | 66 |
| March 17, 2022 | Health Canada | - Allergic Rhinitis - Serum IgE - IgE - Skin Prick Test - Scratch Test - Puncture Test | 67 |
| March 18, 2022 | National Institutes of Health | - Allergic Rhinitis - Serum IgE - IgE - Skin Prick Test - Scratch Test - Puncture Test | 100 |
| March 23, 2022 | European Academy of Allergy and Clinical Immunology | Via Google: "allergic rhinitis" ("serum igE" OR "igE" OR "skin prick test" OR "puncture test" OR "scratch test") site:https://www.eaaci.org/ | 26 |
| March 23, 2022 | Australasian Society of Clinical Immunology and Allergy | Via Google: "allergic rhinitis" ("serum igE" OR "igE" OR "skin prick test" OR "puncture test" OR "scratch test") site:https://www.allergy.org.au/ | 186 |
| March 23, 2022 | American Academy of Allergy, Asthma & Immunology | Via Google: "allergic rhinitis" ("serum igE" OR "igE" OR "skin prick test" OR "puncture test" OR "scratch test") site:https://www.aaaai.org/ | 153 |
| March 25, 2022 | Google [verbatim search] (first 5 pages) | "allergic rhinitis" ("serum igE" OR "igE" OR "skin prick test" OR "puncture test" OR "scratch test") "systematic review" | 50 |
| March 25, 2022 | Google [verbatim search] (first 5 pages) | "allergic rhinitis" ("serum igE" OR "igE" OR "skin prick test" OR "puncture test" OR "scratch test") ("practice guideline" OR "clinical guideline") | 50 |
| March 28, 2022 | British Society for Allergy & Clinical Immunology | - Allergic Rhinitis - Skin Prick Test - Puncture Test - Scratch Test - Serum IgE - IgE | 1 |
| March 30, 2022 | Google (first 5 pages) | - Allergic Rhinitis Consensus | 50 |

# Appendix 2

## Research Question 2: When taking into account the preferences of the patient and the prescriber (stakeholder engagement) should second generation antihistamine or intranasal corticosteroid be first line?

Due to the amount of available literature on second generation antihistamines and intranasal corticosteroids as treatment for allergic rhinitis, this search was conducted in different iterations, focusing on three types of high-quality evidence: clinical practice guidelines, systematic reviews, and randomized controlled trials. As there was plenty of available evidence in these three types of publications, this search did not include other levels of evidence that were not prioritized for this project, such as observational studies. In order to identify literature in each of these categories, the search created to identify literature on this topic in the Medline database was combined with a modified version of established and tested search filters from CADTH for each publication type. Each search filter had been designed to identify either clinical practice guidelines, systematic reviews and meta-analyses or randomized controlled trials.

## Inclusion/Exclusion Criteria

A total of 164 results were returned in the database searches and 541 results were returned from searching the Internet and online resources. A reviewer screened these results for relevance to the research question and applied the inclusion and exclusion criteria for this project. Duplicate results from different searches were removed.

The inclusion criteria were as follows:

- Adult or pediatric participants with allergic rhinitis
- Focus on oral second-generation antihistamines or intranasal corticosteroids for the treatment of allergic rhinitis, ideally a comparison of the two treatments. Third or newer generation antihistamines were not excluded from the search results.
- Comparisons of either oral second-generation antihistamines or intranasal corticosteroids with other treatments were included in the search results
- Comparisons among types of second-generation oral antihistamines or intranasal corticosteroids were included in the search results
- Clinical practice guidelines, systematic reviews or meta-analyses, or randomized controlled trials
- English language
- Published in the year 2016 or later
- Human subject studies

Exclusion criteria for the search were as follows:

- Articles focused only on first-generation antihistamines or intranasal antihistamines
- Articles focused on food allergy and not environmental allergens
- Articles focused on local allergic rhinitis, non-allergic rhinitis, occupational allergic rhinitis or other respiratory conditions such as asthma that did not also include allergic rhinitis patients.

## Medline Search Strategies

Database: Ovid MEDLINE(R) ALL <1946 to May 03, 2022>

Search Strategy (Guidelines)

--------------------------------------------------------------------------------

1 exp Rhinitis, Allergic/ (22922)

2 ((seasonal or intermittent or persistent or perennial or ragweed or grass or birch or house dust mite or mold or dander or cat or aeroallergen) adj4 (allergic rhinitis or allergic rhinitides)).mp. (3668)

3 hay fever.mp. (3874)

4 1 or 2 or 3 (24757)

5 Histamine H1 Antagonists, Non-Sedating/ (1096)

6 ((second or new* or 2nd) adj generation adj (antihistamine* or AH or H1A or H1R antagonists or H1 receptor antagonists or H1 antagonists)).mp. (609)

7 (non-sedat* adj (antihistamine* or AH or H1A or H1R antagonists or H1 receptor antagonists or H1 antagonists)).mp. (222)

8 (Alavert or Allegra or Aller-Tec or Cetirizine or Zyrtec or Clarinex or Claritin or Desloratadine or Fexofenadine or Levocetirizine or Loratadine or Mucinex or Pediacare or Qlearquil or Quzyttir or Wal-Zyr or Xyzal or Azelastine or Astelin).mp. (5104)

9 5 or 6 or 7 or 8 (5726)

10 ((intranasal adj (glucocorticoid* or corticosteroid*)) or INCS).mp. [mp=title, abstract, original title, name of substance word, subject heading word, floating sub-heading word, keyword heading word, organism supplementary concept word, protocol supplementary concept word, rare disease supplementary concept word, unique identifier, synonyms] (1202)

11 (Beconase or Flonase or Nasacort or Nasalide or Nasarel or Nasonex or Omnaris or Rhinocort or Tri-Nasal or Vancenase or Veramyst or Zetonna or Apo-Beclomethasone Aq or Beclomethasone Aq or Gen-Budesonide Aq or Med Beclomethasone Aq or Nu-Beclomethasone or Nu-Flunisolide or Ratio-Beclomethasone Aq).mp. (131)

12 10 or 11 (1317)

13 9 or 12 (6913)

14 4 and 13 (1790)

15 limit 14 to (english language and humans and yr="2016 -Current") (199)

16 exp clinical pathway/ (7497)

17 exp clinical protocol/ (184704)

18 clinical protocols/ (29690)

19 exp consensus/ (18466)

20 exp consensus development conference/ (12610)

21 exp consensus development conferences as topic/ (2997)

22 critical pathways/ (7497)

23 exp guideline/ (37002)

24 guidelines as topic/ (42006)

25 exp practice guideline/ (29795)

26 practice guidelines as topic/ (127287)

27 health planning guidelines/ (4167)

28 Clinical Decision Rules/ (867)

29 (guideline or practice guideline or consensus development conference or consensus development conference, NIH).pt. (46851)

30 (position statement* or policy statement* or practice parameter* or best practice*).ti,ab,kf,kw. (41324)

31 (standards or guideline or guidelines).ti,kf,kw. (126380)

32 ((practice or treatment* or clinical) adj guideline*).ab. (48014)

33 (CPG or CPGs).ti. (6192)

34 consensus*.ti,kf,kw. (31551)

35 consensus*.ab. /freq=2 {Including Related Terms} (1)

36 ((critical or clinical or practice) adj2 (path or paths or pathway or pathways or protocol*)).ti,ab,kf,kw. (24231)

37 recommendat*.ti,kf,kw. or guideline recommendation*.ab. (53399)

38 (care adj2 (standard or path or paths or pathway or pathways or map or maps or plan or plans)).ti,ab,kf,kw. (74217)

39 (algorithm* adj2 (screening or examination or test or tested or testing or assessment* or diagnosis or diagnoses or diagnosed or diagnosing)).ti,ab,kf,kw. (9329)

40 (algorithm* adj2 (pharmacotherap* or chemotherap* or chemotreatment* or therap* or treatment* or intervention*)).ti,ab,kf,kw. (11836)

41 (guideline* or standards or consensus* or recommendat*).au. (550)

42 (guideline* or standards or consensus* or recommendat*).ca. (1179)

43 or/16-42 (691001)

44 15 and 43 (17)

***************************

Database: Ovid MEDLINE(R) ALL <1946 to May 03, 2022>

Search Strategy (Systematic Reviews)

--------------------------------------------------------------------------------

1 exp Rhinitis, Allergic/ (22922)

2 ((seasonal or intermittent or persistent or perennial or ragweed or grass or birch or house dust mite or mold or dander or cat or aeroallergen) adj4 (allergic rhinitis or allergic rhinitides)).mp. (3668)

3 hay fever.mp. (3874)

4 1 or 2 or 3 (24757)

5 Histamine H1 Antagonists, Non-Sedating/ (1096)

6 ((second or new* or 2nd) adj generation adj (antihistamine* or AH or H1A or H1R antagonists or H1 receptor antagonists or H1 antagonists)).mp. (609)

7 (non-sedat* adj (antihistamine* or AH or H1A or H1R antagonists or H1 receptor antagonists or H1 antagonists)).mp. (222)

8 (Alavert or Allegra or Aller-Tec or Cetirizine or Zyrtec or Clarinex or Claritin or Desloratadine or Fexofenadine or Levocetirizine or Loratadine or Mucinex or Pediacare or Qlearquil or Quzyttir or Wal-Zyr or Xyzal or Azelastine or Astelin).mp. (5104)

9 5 or 6 or 7 or 8 (5726)

10 ((intranasal adj (glucocorticoid* or corticosteroid*)) or INCS).mp. [mp=title, abstract, original title, name of substance word, subject heading word, floating sub-heading word, keyword heading word, organism supplementary concept word, protocol supplementary concept word, rare disease supplementary concept word, unique identifier, synonyms] (1202)

11 (Beconase or Flonase or Nasacort or Nasalide or Nasarel or Nasonex or Omnaris or Rhinocort or Tri-Nasal or Vancenase or Veramyst or Zetonna or Apo-Beclomethasone Aq or Beclomethasone Aq or Gen-Budesonide Aq or Med Beclomethasone Aq or Nu-Beclomethasone or Nu-Flunisolide or Ratio-Beclomethasone Aq).mp. (131)

12 10 or 11 (1317)

13 9 or 12 (6913)

14 4 and 13 (1790)

15 limit 14 to (english language and humans and yr="2016 -Current") (199)

16 (systematic review or meta-analysis).pt. (271589)

17 meta-analysis/ or systematic review/ or systematic reviews as topic/ or meta-analysis as topic/ or "meta analysis (topic)"/ or "systematic review (topic)"/ or exp technology assessment, biomedical/ or network meta-analysis/ (307486)

18 ((systematic* adj3 (review* or overview*)) or (methodologic* adj3 (review* or overview*))).ti,ab,kf,kw. (268658)

19 ((quantitative adj3 (review* or overview* or synthes*)) or (research adj3 (integrati* or overview*))).ti,ab,kf,kw. (13753)

20 ((integrative adj3 (review* or overview*)) or (collaborative adj3 (review* or overview*)) or (pool* adj3 analy*)).ti,ab,kf,kw. (34355)

21 (data synthes* or data extraction* or data abstraction*).ti,ab,kf,kw. (35257)

22 (handsearch* or hand search*).ti,ab,kf,kw. (10464)

23 (mantel haenszel or peto or der simonian or dersimonian or fixed effect* or latin square*).ti,ab,kf,kw. (31941)

24 (met analy* or metanaly* or technology assessment* or HTA or HTAs or technology overview* or technology appraisal*).ti,ab,kf,kw. (11106)

25 (meta regression* or metaregression*).ti,ab,kf,kw. (12430)

26 (meta-analy* or metaanaly* or systematic review* or biomedical technology assessment* or bio-medical technology assessment*).mp,hw. (404974)

27 (medline or cochrane or pubmed or medlars or embase or cinahl).ti,ab,hw. (294298)

28 (cochrane or (health adj2 technology assessment) or evidence report).jw. (20783)

29 (comparative adj3 (efficacy or effectiveness)).ti,ab,kf,kw. (16001)

30 (outcomes research or relative effectiveness).ti,ab,kf,kw. (10589)

31 ((indirect or indirect treatment or mixed-treatment or bayesian) adj3 comparison*).ti,ab,kf,kw. (3937)

32 (multi* adj3 treatment adj3 comparison*).ti,ab,kf,kw. (274)

33 (mixed adj3 treatment adj3 (meta-analy* or metaanaly*)).ti,ab,kf,kw. (174)

34 umbrella review*.ti,ab,kf,kw. (943)

35 (multi* adj2 paramet* adj2 evidence adj2 synthesis).ti,ab,kw,kf. (13)

36 (multiparamet* adj2 evidence adj2 synthesis).ti,ab,kw,kf. (17)

37 (multi-paramet* adj2 evidence adj2 synthesis).ti,ab,kw,kf. (11)

38 or/16-37 (599880)

39 15 and 38 (38)

***************************

Database: Ovid MEDLINE(R) ALL <1946 to May 03, 2022>

Search Strategy (Randomized Controlled Trials)

--------------------------------------------------------------------------------

1 exp Rhinitis, Allergic/ (22922)

2 ((seasonal or intermittent or persistent or perennial or ragweed or grass or birch or house dust mite or mold or dander or cat or aeroallergen) adj4 (allergic rhinitis or allergic rhinitides)).mp. (3668)

3 hay fever.mp. (3874)

4 1 or 2 or 3 (24757)

5 Histamine H1 Antagonists, Non-Sedating/ (1096)

6 ((second or new* or 2nd) adj generation adj (antihistamine* or AH or H1A or H1R antagonists or H1 receptor antagonists or H1 antagonists)).mp. (609)

7 (non-sedat* adj (antihistamine* or AH or H1A or H1R antagonists or H1 receptor antagonists or H1 antagonists)).mp. (222)

8 (Alavert or Allegra or Aller-Tec or Cetirizine or Zyrtec or Clarinex or Claritin or Desloratadine or Fexofenadine or Levocetirizine or Loratadine or Mucinex or Pediacare or Qlearquil or Quzyttir or Wal-Zyr or Xyzal or Azelastine or Astelin).mp. (5104)

9 5 or 6 or 7 or 8 (5726)

10 ((intranasal adj (glucocorticoid* or corticosteroid*)) or INCS).mp. [mp=title, abstract, original title, name of substance word, subject heading word, floating sub-heading word, keyword heading word, organism supplementary concept word, protocol supplementary concept word, rare disease supplementary concept word, unique identifier, synonyms] (1202)

11 (Beconase or Flonase or Nasacort or Nasalide or Nasarel or Nasonex or Omnaris or Rhinocort or Tri-Nasal or Vancenase or Veramyst or Zetonna or Apo-Beclomethasone Aq or Beclomethasone Aq or Gen-Budesonide Aq or Med Beclomethasone Aq or Nu-Beclomethasone or Nu-Flunisolide or Ratio-Beclomethasone Aq).mp. (131)

12 10 or 11 (1317)

13 9 or 12 (6913)

14 4 and 13 (1790)

15 limit 14 to (english language and humans and yr="2016 -Current") (199)

16 (Randomized Controlled Trial or Controlled Clinical Trial or Pragmatic Clinical Trial or Equivalence Trial or Clinical Trial, Phase III).pt. (662761)

17 Randomized Controlled Trial/ (568175)

18 exp Randomized Controlled Trials as Topic/ (159228)

19 "Randomized Controlled Trial (topic)"/ (0)

20 Controlled Clinical Trial/ (94879)

21 exp Controlled Clinical Trials as Topic/ (164905)

22 "Controlled Clinical Trial (topic)"/ (0)

23 Randomization/ (106859)

24 Random Allocation/ (106859)

25 Double-Blind Method/ (171766)

26 Double Blind Procedure/ (0)

27 Double-Blind Studies/ (171766)

28 Single-Blind Method/ (31920)

29 Single Blind Procedure/ (0)

30 Single-Blind Studies/ (31920)

31 Placebos/ (35923)

32 Placebo/ (0)

33 Control Groups/ (1833)

34 Control Group/ (1833)

35 (random* or sham or placebo*).ti,ab,hw,kf,kw. (1694430)

36 ((singl* or doubl*) adj (blind* or dumm* or mask*)).ti,ab,hw,kf,kw. (257238)

37 ((tripl* or trebl*) adj (blind* or dumm* or mask*)).ti,ab,hw,kf,kw. (1366)

38 (control* adj3 (study or studies or trial* or group*)).ti,ab,kf,kw. (1132585)

39 (Nonrandom* or non random* or non-random* or quasi-random* or quasirandom*).ti,ab,hw,kf,kw. (50577)

40 allocated.ti,ab,hw. (76993)

41 ((open label or open-label) adj5 (study or studies or trial*)).ti,ab,hw,kf,kw. (40986)

42 ((equivalence or superiority or non-inferiority or noninferiority) adj3 (study or studies or trial*)).ti,ab,hw,kf,kw. (10719)

43 (pragmatic study or pragmatic studies).ti,ab,hw,kf,kw. (524)

44 ((pragmatic or practical) adj3 trial*).ti,ab,hw,kf,kw. (6892)

45 ((quasiexperimental or quasi-experimental) adj3 (study or studies or trial*)).ti,ab,hw,kf,kw. (10352)

46 (phase adj3 (III or "3") adj3 (study or studies or trial*)).ti,hw,kf,kw. (33341)

47 or/16-46 (2430963)

48 15 and 47 (108)

***************************

## Cochrane Database of Systematic Reviews Search Strategy (May 2, 2022)

S1 Allergic Rhinitis

S2 ((seasonal or intermittent or persistent or perennial or ragweed or grass or birch or house dust mite or mold or dander or cat or aeroallergen) N4 (allergic rhinitis or allergic rhinitides))

S3 hay fever

S4 S1 OR S2 OR S3

S5 oral antihistamines

S6 ((second or new* or 2nd) N2 generation N2 (antihistamine* or AH or H1A or H1R antagonists or H1 receptor antagonists or H1 antagonists))

S7 (non-sedat* N2 (antihistamine* or AH or H1A or H1R antagonists or H1 receptor antagonists or H1 antagonists))

S8 (Alavert or Allegra or Aller-Tec or Cetirizine or Zyrtec or Clarinex or Claritin or Desloratadine or Fexofenadine or Levocetirizine or Loratadine or Mucinex or Pediacare or Qlearquil or Quzyttir or Wal-Zyr or Xyzal or Azelastine or Astelin)

S9 S5 OR S6 OR S7 OR S8

S10 ((intranasal N2 (glucocorticoid* or corticosteroid*)) or INCS)

S11 (Beconase or Flonase or Nasacort or Nasalide or Nasarel or Nasonex or Omnaris or Rhinocort or Tri-Nasal or Vancenase or Veramyst or Zetonna or Apo-Beclomethasone Aq or Beclomethasone Aq or Gen-Budesonide Aq or Med Beclomethasone Aq or Nu-Beclomethasone or Nu-Flunisolide or Ratio-Beclomethasone Aq)

S12 S10 OR S11

S13 S9 OR S12

S14 S4 AND S13 (9)

## Internet Search Strategies

| Date | Repository | Search terms | # Results |
| --- | --- | --- | --- |
| April 25, 2022 | Google (first 5 pages) | - antihistamine AND guideline AND allergic rhinitis - intranasal corticosteroid AND guideline AND allergic rhinitis - antihistamines AND guidance AND allergic rhinitis - intranasal corticosteroids AND guidance AND allergic rhinitis - antihistamines AND consensus AND allergic rhinitis - intranasal corticosteroids AND consensus AND allergic rhinitis - intranasal corticosteroids AND systematic reviews AND allergic rhinitis - intranasal corticosteroids AND meta analyses AND allergic rhinitis - antihistamines AND meta analyses AND allergic rhinitis - antihistamines AND rct AND allergic rhinitis - intranasal corticosteroids AND rct AND allergic rhinitis | 100 |
| April 26, 2022 | CMA Infobase | - Allergic Rhinitis - antihistamines - intranasal corticosteroids | 7 |
| April 26, 2022 | NICE | - Allergic Rhinitis - antihistamine - intranasal corticosteroid | 36 |
| April 26, 2022 | Health Canada | - Allergic Rhinitis - antihistamine allergic rhinitis - intranasal corticosteroid | 246 |
| April 26, 2022 | National Institutes of Health | - Allergic Rhinitis - Allergic rhinitis antihistamines - Allergic rhinitis intranasal corticosteroids | 100 |
| April 26, 2022 | European Academy of Allergy and Clinical Immunology | Via Google: "allergic rhinitis" ("antihistamines OR intranasal corticosteroid") site:https://www.eaaci.org/ | 2 |
| April 26, 2022 | Australasian Society of Clinical Immunology and Allergy | Via Google: "allergic rhinitis" ("antihistamines OR intranasal corticosteroid") site:https://www.allergy.org.au/ | 40 |
| April 26, 2022 | American Academy of Allergy, Asthma & Immunology | Via Google: "allergic rhinitis" ("antihistamines OR intranasal corticosteroid") site:https://www.aaaai.org/ | 10 |

# Appendix 3

## Research question 3: Is a combination intranasal antihistamine/intranasal corticosteroid formulation superior to intranasal corticosteroid plus oral antihistamine? Do they become equivalent after prolonged use?

Due to the small number of published studies in the area, study type filters were not applied to the database searches for this topic and screening for relevance and study type was done manually.

## Inclusion/Exclusion Criteria

A total of 54 results were returned in the database searches and 1439 results were returned from searching the Internet and online resources. A reviewer screened these results for relevance to the research question and applied the inclusion and exclusion criteria for this project. Duplicate results from different searches were removed.

The inclusion criteria were as follows:

- Adult or pediatric participants with allergic rhinitis
- Focused on combination intranasal antihistamine and intranasal corticosteroid or use of intranasal corticosteroids plus oral antihistamines for the treatment of allergic rhinitis. Studies where both treatment options are discussed are the ideal.
- Articles where either treatment option is compared to other treatments outside the clinical question were eligible for inclusion.
- Clinical practice guidelines, systematic reviews or meta-analyses, or randomized controlled trials were prioritized though other original studies of interest were eligible for inclusion.
- English language
- Published in the year 2016 or later
- Human subject studies

Exclusion criteria was as follows:

- Articles focused on monotherapy rather than combination therapy
- Articles focused on food allergy and not environmental allergens
- Articles focused on local allergic rhinitis, non-allergic rhinitis, occupational allergic rhinitis or other respiratory conditions such as asthma, that did not also include allergic rhinitis patients.

## Medline Search Strategies

Database: Ovid MEDLINE(R) ALL <1946 to May 04, 2022>

Search Strategy:

--------------------------------------------------------------------------------

1 exp Rhinitis, Allergic/ (22904)

2 ((seasonal or intermittent or persistent or perennial or ragweed or grass or birch or house dust mite or mold or dander or cat or aeroallergen) adj4 (allergic rhinitis or allergic rhinitides)).mp. (3665)

3 hay fever.mp. (3874)

4 1 or 2 or 3 (24739)

5 Histamine H1 Antagonists, Non-Sedating/ (1095)

6 ((second or new* or 2nd) adj generation adj (antihistamine* or AH or H1A or H1R antagonists or H1 receptor antagonists or H1 antagonists)).mp. (609)

7 (non-sedat* adj (antihistamine* or AH or H1A or H1R antagonists or H1 receptor antagonists or H1 antagonists)).mp. (222)

8 (Alavert or Allegra or Aller-Tec or Cetirizine or Zyrtec or Clarinex or Claritin or Desloratadine or Fexofenadine or Levocetirizine or Loratadine or Mucinex or Pediacare or Qlearquil or Quzyttir or Wal-Zyr or Xyzal or Azelastine or Astelin).mp. (5103)

9 5 or 6 or 7 or 8 (5725)

10 (intranasal corticosteroids or INCS).mp. (944)

11 (Beconase or Flonase or Nasacort or Nasalide or Nasarel or Nasonex or Omnaris or Rhinocort or Tri-Nasal or Vancenase or Veramyst or Zetonna or Apo-Beclomethasone Aq or Beclomethasone Aq or Gen-Budesonide Aq or Med Beclomethasone Aq or Nu-Beclomethasone or Nu-Flunisolide or Ratio-Beclomethasone Aq).mp. (131)

12 10 or 11 (1067)

13 4 and 9 and 12 (85)

14 ((Combination or combined) adj10 ((nasal or intranasal) adj (steroid or corticosteroid)) adj10 antihistamine).mp. (20)

15 Dymista.mp. (26)

16 Ryaltris.mp. (0)

17 MP-AzeFlu.mp. (33)

18 (fluticasone adj3 azelastine).mp. (61)

19 (Mometasone adj3 Olopatadine).mp. (10)

20 or/14-19 (112)

21 4 and 20 (82)

22 13 or 21 (149)

23 limit 22 to (english language and humans and yr="2016 -Current") (51)

***************************

## Cochrane Databases Search Strategy

S1 Allergic Rhinitis

S2 ((seasonal or intermittent or persistent or perennial or ragweed or grass or birch or house dust mite or mold or dander or cat or aeroallergen) N4 (allergic rhinitis or allergic rhinitides))

S3 hay fever

S4 S1 OR S2 OR S3

S5 oral antihistamines

S6 ((second or new* or 2nd) n1 generation n1 (antihistamine* or AH or H1A or H1R antagonists or H1 receptor antagonists or H1 antagonists))

S7 (non-sedat* n2 (antihistamine* or AH or H1A or H1R antagonists or H1 receptor antagonists or H1 antagonists))

S8 (Alavert or Allegra or Aller-Tec or Cetirizine or Zyrtec or Clarinex or Claritin or Desloratadine or Fexofenadine or Levocetirizine or Loratadine or Mucinex or Pediacare or Qlearquil or Quzyttir or Wal-Zyr or Xyzal or Azelastine or Astelin)

S9 S5 OR S6 OR S7 OR S8

S10 (intranasal corticosteroids or INCS)

S11 (Beconase or Flonase or Nasacort or Nasalide or Nasarel or Nasonex or Omnaris or Rhinocort or Tri-Nasal or Vancenase or Veramyst or Zetonna or Apo-Beclomethasone Aq or Beclomethasone Aq or Gen-Budesonide Aq or Med Beclomethasone Aq or Nu-Beclomethasone or Nu-Flunisolide or Ratio-Beclomethasone Aq)

S12 S10 OR S11

S13 S4 AND S9 AND S12

S14 ((Combination or combined) N10 ((nasal or intranasal) N1 (steroid or corticosteroid)) N10 antihistamine)

S15 Dymista

S16 Ryaltris

S17 MP-AzeFlu

S18 (fluticasone N3 azelastine)

S19 (Mometasone N3 Olopatadine)

S20 S14 OR S15 OR S16 OR S17 OR S18 OR S19

S21 S4 AND S20

S22 S13 OR S21 (3)

## Internet Search Strategies

| Date | Repository | Search terms | # Results |
| --- | --- | --- | --- |
| May 6, 2022 | Google (first 5 pages) | - Combination antihistamine corticosteroid AND Allergic Rhinitis AND guidance - Combination antihistamine corticosteroid AND Allergic Rhinitis AND guideline - Combination antihistamine corticosteroid AND Allergic Rhinitis AND consensus - Combination antihistamine corticosteroid AND Allergic Rhinitis AND systematic review - Combination antihistamine corticosteroid AND Allergic Rhinitis AND meta analyses - Combination antihistamine corticosteroid AND Allergic Rhinitis AND randomized controlled trials - antihistamine AND guideline AND allergic rhinitis - intranasal corticosteroid AND guideline AND allergic rhinitis - antihistamines AND guidance AND allergic rhinitis - intranasal corticosteroids AND guidance AND allergic rhinitis - antihistamines AND consensus AND allergic rhinitis - intranasal corticosteroids AND consensus AND allergic rhinitis - intranasal corticosteroids AND systematic reviews AND allergic rhinitis - intranasal corticosteroids AND meta analyses AND allergic rhinitis - antihistamines AND meta analyses AND allergic rhinitis - antihistamines AND rct AND allergic rhinitis - intranasal corticosteroids AND rct AND allergic rhinitis | 850 |
| May 6, 2022 | CMA Infobase | - Allergic Rhinitis - antihistamine - intranasal corticosteroids | 7 |
| May 6, 2022 | NICE | - Allergic Rhinitis - antihistamine - intranasal corticosteroid | 42 |
| May 6, 2022 | Health Canada | - Allergic Rhinitis - antihistamine allergic rhinitis - intranasal corticosteroid - combination antihistamines corticosteroids | 287 |
| May 6, 2022 | National Institutes of Health | - Allergic Rhinitis - Allergic rhinitis antihistamines - Allergic rhinitis intranasal corticosteroids - combination antihistamines corticosteroids allergic rhinitis | 200 |
| May 6, 2022 | European Academy of Allergy and Clinical Immunology | Via Google: "allergic rhinitis" ("antihistamines OR intranasal corticosteroid") site:<https://www.eaaci.org/>  Via Google: "allergic rhinitis" ("combination antihistamines intranasal corticosteroid") site:<https://www.eaaci.org/> | 2 |
| May 6, 2022 | Australasian Society of Clinical Immunology and Allergy | Via Google: "allergic rhinitis" ("combination antihistamines intranasal corticosteroid") site:<https://www.allergy.org.au/>  Via Google: "allergic rhinitis" ("antihistamines OR intranasal corticosteroid") site:https://www.allergy.org.au/ | 41 |
| May 6, 2022 | American Academy of Allergy, Asthma & Immunology | Via Google: "allergic rhinitis" ("combination antihistamines intranasal corticosteroid") site:<https://www.aaaai.org/>  Via Google: "allergic rhinitis" ("antihistamines OR intranasal corticosteroid") site:<https://www.aaaai.org/> | 10 |

# Appendix 4

## Research Question 4: Do leukotriene receptor antagonists (LTRA) have a greater benefit than oral antihistamines (OAH) in allergic rhinitis for some symptoms to justify a therapeutic trial in those who cannot tolerate intranasal corticosteroids?

## Inclusion/Exclusion Criteria

A total of 167 results were returned in the database searches and 1329 results were returned from searching the Internet and online resources. A reviewer screened these results for relevance to the research question and applied the inclusion and exclusion criteria for this project. Duplicate results from different searches were removed. The inclusion criteria was as follows:

- Adult or pediatric participants with allergic rhinitis
- Focus on leukotriene receptor antagonists for the treatment of allergic rhinitis in comparison to other interventions such as oral antihistamines or placebo
- Clinical practice guidelines, systematic reviews or meta-analyses, or randomized controlled trials were prioritized though other original studies of interest were eligible for inclusion.
- English language publications
- Published in the year 2016 or later
- Human subject studies

Exclusion criteria for the search was as follows:

- Articles focused on leukotriene receptor antagonists only in the context of asthma treatment
- Articles focused on local allergic rhinitis, non-allergic rhinitis, occupational allergic rhinitis or other respiratory conditions such as asthma, that did not also include allergic rhinitis patients.

## Medline Search Strategies

Database: Ovid MEDLINE(R) ALL <1946 to May 09, 2022>

Search Strategy:

--------------------------------------------------------------------------------

1 exp Rhinitis, Allergic/ (22913)

2 ((seasonal or intermittent or persistent or perennial or ragweed or grass or birch or house dust mite or mold or dander or cat or aeroallergen) adj4 (allergic rhinitis or allergic rhinitides)).mp. (3667)

3 hay fever.mp. (3874)

4 1 or 2 or 3 (24749)

5 Leukotriene Antagonists/ (3210)

6 (leukotriene adj2 (receptor antagonist* or modifier* or synthesis inhibitor*)).mp. (2444)

7 Antileukotriene.mp. (202)

8 montelukast.mp. (2759)

9 or/5-8 (5878)

10 Histamine H1 Antagonists, Non-Sedating/ (1096)

11 ((second or new* or 2nd) adj generation adj (antihistamine* or AH or H1A or H1R antagonists or H1 receptor antagonists or H1 antagonists)).mp. (609)

12 (non-sedat* adj (antihistamine* or AH or H1A or H1R antagonists or H1 receptor antagonists or H1 antagonists)).mp. (222)

13 (Alavert or Allegra or Aller-Tec or Cetirizine or Zyrtec or Clarinex or Claritin or Desloratadine or Fexofenadine or Levocetirizine or Loratadine or Mucinex or Pediacare or Qlearquil or Quzyttir or Wal-Zyr or Xyzal or Azelastine or Astelin).mp. (5106)

14 or/10-13 (5728)

15 9 or 14 (11288)

16 4 and 15 (1610)

17 limit 16 to (english language and humans and yr="2016 -Current") (161)

***************************

## Cochrane Databases Search Strategy

S1 Allergic Rhinitis

S2 ((seasonal or intermittent or persistent or perennial or ragweed or grass or birch or house dust mite or mold or dander or cat or aeroallergen) N4 (allergic rhinitis or allergic rhinitides))

S3 hay fever

S4 S1 OR S2 OR S3

S5 Leukotriene Antagonists

S6 (leukotriene N2 (receptor antagonist* or modifier* or synthesis inhibitor*))

S7 Antileukotriene

S8 montelukast

S9 S5 OR S6 OR S7 OR S8

S10 oral antihistamines

S11 ((second or new* or 2nd) N2 generation N2 (antihistamine* or AH or H1A or H1R antagonists or H1 receptor antagonists or H1 antagonists))

S12 (non-sedat* N2 (antihistamine* or AH or H1A or H1R antagonists or H1 receptor antagonists or H1 antagonists))

S13 (Alavert or Allegra or Aller-Tec or Cetirizine or Zyrtec or Clarinex or Claritin or Desloratadine or Fexofenadine or Levocetirizine or Loratadine or Mucinex or Pediacare or Qlearquil or Quzyttir or Wal-Zyr or Xyzal or Azelastine or Astelin)

S14 S10 OR S11 OR S12 OR S13

S15 S9 OR S14

S16 S4 AND S15 (6)

## Internet Search Strategies

| Date | Repository | Search terms | # Results |
| --- | --- | --- | --- |
| May 9, 2022 | Google (first 5 pages) | - antihistamine AND guideline AND allergic rhinitis - antihistamines AND guidance AND allergic rhinitis - antihistamines AND consensus AND allergic rhinitis - antihistamines AND meta analyses AND allergic rhinitis - antihistamines AND rct AND allergic rhinitis - leukotriene receptor antagonist allergic rhinitis - LTRA allergic rhinitis - Montelukast allergic rhinitis - leukotriene receptor antagonist allergic rhinitis systematic review - LTRA allergic rhinitis systematic review - Montelukast allergic rhinitis systematic review - leukotriene receptor antagonist allergic rhinitis rct - LTRA allergic rhinitis rct - Montelukast allergic rhinitis rct | 550 |
| May 9, 2022 | CMA Infobase | - Allergic Rhinitis - Antihistamines - leukotriene receptor antagonist allergic rhinitis - LTRA allergic rhinitis - Montelukast allergic rhinitis | 7 |
| May 9, 2022 | NICE | - Allergic Rhinitis - Antihistamine - leukotriene receptor antagonist allergic rhinitis - LTRA allergic rhinitis - Montelukast allergic rhinitis | 52 |
| May 9, 2022 | Health Canada | - Allergic Rhinitis - antihistamine allergic rhinitis - leukotriene receptor antagonist - LTRA - Montelukast | 340 |
| May 9, 2022 | National Institutes of Health | - Allergic Rhinitis - Allergic rhinitis antihistamines - leukotriene receptor antagonist allergic rhinitis - LTRA allergic rhinitis - Montelukast allergic rhinitis | 250 |
| May 10, 2022 | European Academy of Allergy and Clinical Immunology | Via Google: "allergic rhinitis" ("antihistamines") site:<https://www.eaaci.org/>  "allergic rhinitis" ("montelukast") site:<https://www.eaaci.org/>  "allergic rhinitis" ("leukotriene receptor antagonist") site:<https://www.eaaci.org/>  "allergic rhinitis" ("LTRA") site:<https://www.eaaci.org/> | 5 |
| May 10, 2022 | Australasian Society of Clinical Immunology and Allergy | Via Google: "allergic rhinitis" ("antihistamines") site:<https://www.allergy.org.au/>  "allergic rhinitis" ("leukotriene receptor antagonist") site:<https://www.allergy.org.au/>  "allergic rhinitis" ("LTRA") site:<https://www.allergy.org.au/>  "allergic rhinitis" ("montelukast") site:https://www.allergy.org.au/ | 55 |
| May 10, 2022 | American Academy of Allergy, Asthma & Immunology | Via Google: "allergic rhinitis" ("antihistamines") site:<https://www.aaaai.org/>  "allergic rhinitis" ("leukotriene receptor antagonist") site:<https://www.aaaai.org/>  "allergic rhinitis" ("LTRA") site:<https://www.aaaai.org/>  "allergic rhinitis" ("montelukast") site:<https://www.aaaai.org/> | 70 |

# Appendix 5

## Research Question 5: Should sublingual immunotherapy (SLIT) tablets be considered first-line immunotherapeutic options over subcutaneous immunotherapy (SCIT) based on the evidence of efficacy?

## Inclusion/Exclusion Criteria

A total of 284 results were returned in the database searches and 519 results were returned from searching the Internet and online resources. A reviewer screened these results for relevance to the research question and applied the inclusion and exclusion criteria for this project. Duplicate results from different searches were removed. The inclusion criteria were as follows:

- Adult or pediatric participants with allergic rhinitis
- Treatment with SCIT or SLIT tablets
- Clinical practice guidelines, systematic reviews or meta-analyses, or randomized controlled trials were prioritized
- Clinical practice guidelines, systematic reviews or randomized controlled trials of all treatment lengths
- Other study types reporting on treatment lengths of three years or more
- Papers such as systematic reviews that included data from treatment with SLIT drops as well as SLIT tablets were included with an identifying note.
- English language
- Published in the year 2016 or later
- Human subject studies

Exclusion criteria for the search was as follows:

- Studies focused on treatment with only SLIT drops and not tablets
- Articles focused on food allergy and not environmental allergens
- Articles focused on local allergic rhinitis, non-allergic rhinitis, occupational allergic rhinitis or other respiratory conditions such as asthma, that did not also include allergic rhinitis patients.

## Medline Search Strategies

Database: Ovid MEDLINE(R) ALL <1946 to May 18, 2022>

Search Strategy:

--------------------------------------------------------------------------------

1 exp Rhinitis, Allergic/ (22940)

2 ((seasonal or intermittent or persistent or perennial or ragweed or grass or birch or house dust mite or mold or dander or cat or aeroallergen) adj4 (allergic rhinitis or allergic rhinitides)).mp. (3667)

3 hay fever.mp. (3875)

4 1 or 2 or 3 (24775)

5 Sublingual Immunotherapy/ (654)

6 ((SLIT or sublingual immunotherap*) adj2 tablet*).mp. (213)

7 5 or 6 (741)

8 Subcutaneous immunotherap*.mp. (992)

9 SCIT.mp. (773)

10 8 or 9 (1286)

11 7 or 10 (1853)

12 4 and 11 (840)

13 limit 12 to (english language and humans and yr="2016 -Current") (371)

14 exp clinical pathway/ (7500)

15 exp clinical protocol/ (184661)

16 clinical protocols/ (29675)

17 exp consensus/ (18497)

18 exp consensus development conference/ (12611)

19 exp consensus development conferences as topic/ (2996)

20 critical pathways/ (7500)

21 exp guideline/ (37033)

22 guidelines as topic/ (42001)

23 exp practice guideline/ (29828)

24 practice guidelines as topic/ (127285)

25 health planning guidelines/ (4164)

26 Clinical Decision Rules/ (868)

27 (guideline or practice guideline or consensus development conference or consensus development conference, NIH).pt. (46885)

28 (position statement* or policy statement* or practice parameter* or best practice*).ti,ab,kf,kw. (41432)

29 (standards or guideline or guidelines).ti,kf,kw. (126495)

30 ((practice or treatment* or clinical) adj guideline*).ab. (48059)

31 (CPG or CPGs).ti. (6195)

32 consensus*.ti,kf,kw. (31591)

33 consensus*.ab. /freq=2 {Including Related Terms} (1)

34 ((critical or clinical or practice) adj2 (path or paths or pathway or pathways or protocol*)).ti,ab,kf,kw. (24274)

35 recommendat*.ti,kf,kw. or guideline recommendation*.ab. (53494)

36 (care adj2 (standard or path or paths or pathway or pathways or map or maps or plan or plans)).ti,ab,kf,kw. (74313)

37 (algorithm* adj2 (screening or examination or test or tested or testing or assessment* or diagnosis or diagnoses or diagnosed or diagnosing)).ti,ab,kf,kw. (9343)

38 (algorithm* adj2 (pharmacotherap* or chemotherap* or chemotreatment* or therap* or treatment* or intervention*)).ti,ab,kf,kw. (11861)

39 (guideline* or standards or consensus* or recommendat*).au. (553)

40 (guideline* or standards or consensus* or recommendat*).ca. (1187)

41 or/14-40 (691533)

42 (systematic review or meta-analysis).pt. (272085)

43 meta-analysis/ or systematic review/ or systematic reviews as topic/ or meta-analysis as topic/ or "meta analysis (topic)"/ or "systematic review (topic)"/ or exp technology assessment, biomedical/ or network meta-analysis/ (307857)

44 ((systematic* adj3 (review* or overview*)) or (methodologic* adj3 (review* or overview*))).ti,ab,kf,kw. (269179)

45 ((quantitative adj3 (review* or overview* or synthes*)) or (research adj3 (integrati* or overview*))).ti,ab,kf,kw. (13759)

46 ((integrative adj3 (review* or overview*)) or (collaborative adj3 (review* or overview*)) or (pool* adj3 analy*)).ti,ab,kf,kw. (34377)

47 (data synthes* or data extraction* or data abstraction*).ti,ab,kf,kw. (35145)

48 (handsearch* or hand search*).ti,ab,kf,kw. (10461)

49 (mantel haenszel or peto or der simonian or dersimonian or fixed effect* or latin square*).ti,ab,kf,kw. (31979)

50 (met analy* or metanaly* or technology assessment* or HTA or HTAs or technology overview* or technology appraisal*).ti,ab,kf,kw. (11109)

51 (meta regression* or metaregression*).ti,ab,kf,kw. (12429)

52 (meta-analy* or metaanaly* or systematic review* or biomedical technology assessment* or bio-medical technology assessment*).mp,hw. (405527)

53 (medline or cochrane or pubmed or medlars or embase or cinahl).ti,ab,hw. (294500)

54 (cochrane or (health adj2 technology assessment) or evidence report).jw. (20744)

55 (comparative adj3 (efficacy or effectiveness)).ti,ab,kf,kw. (16004)

56 (outcomes research or relative effectiveness).ti,ab,kf,kw. (10586)

57 ((indirect or indirect treatment or mixed-treatment or bayesian) adj3 comparison*).ti,ab,kf,kw. (3939)

58 (multi* adj3 treatment adj3 comparison*).ti,ab,kf,kw. (275)

59 (mixed adj3 treatment adj3 (meta-analy* or metaanaly*)).ti,ab,kf,kw. (174)

60 umbrella review*.ti,ab,kf,kw. (962)

61 (multi* adj2 paramet* adj2 evidence adj2 synthesis).ti,ab,kw,kf. (13)

62 (multiparamet* adj2 evidence adj2 synthesis).ti,ab,kw,kf. (17)

63 (multi-paramet* adj2 evidence adj2 synthesis).ti,ab,kw,kf. (11)

64 or/42-63 (600606)

65 (Randomized Controlled Trial or Controlled Clinical Trial or Pragmatic Clinical Trial or Equivalence Trial or Clinical Trial, Phase III).pt. (662932)

66 Randomized Controlled Trial/ (568356)

67 exp Randomized Controlled Trials as Topic/ (159145)

68 "Randomized Controlled Trial (topic)"/ (0)

69 Controlled Clinical Trial/ (94874)

70 exp Controlled Clinical Trials as Topic/ (164824)

71 "Controlled Clinical Trial (topic)"/ (0)

72 Randomization/ (106844)

73 Random Allocation/ (106844)

74 Double-Blind Method/ (171696)

75 Double Blind Procedure/ (0)

76 Double-Blind Studies/ (171696)

77 Single-Blind Method/ (31921)

78 Single Blind Procedure/ (0)

79 Single-Blind Studies/ (31921)

80 Placebos/ (35915)

81 Placebo/ (0)

82 Control Groups/ (1830)

83 Control Group/ (1830)

84 (random* or sham or placebo*).ti,ab,hw,kf,kw. (1695768)

85 ((singl* or doubl*) adj (blind* or dumm* or mask*)).ti,ab,hw,kf,kw. (257202)

86 ((tripl* or trebl*) adj (blind* or dumm* or mask*)).ti,ab,hw,kf,kw. (1371)

87 (control* adj3 (study or studies or trial* or group*)).ti,ab,kf,kw. (1133589)

88 (Nonrandom* or non random* or non-random* or quasi-random* or quasirandom*).ti,ab,hw,kf,kw. (50588)

89 allocated.ti,ab,hw. (77054)

90 ((open label or open-label) adj5 (study or studies or trial*)).ti,ab,hw,kf,kw. (40964)

91 ((equivalence or superiority or non-inferiority or noninferiority) adj3 (study or studies or trial*)).ti,ab,hw,kf,kw. (10714)

92 (pragmatic study or pragmatic studies).ti,ab,hw,kf,kw. (522)

93 ((pragmatic or practical) adj3 trial*).ti,ab,hw,kf,kw. (6898)

94 ((quasiexperimental or quasi-experimental) adj3 (study or studies or trial*)).ti,ab,hw,kf,kw. (10399)

95 (phase adj3 (III or "3") adj3 (study or studies or trial*)).ti,hw,kf,kw. (33313)

96 or/65-95 (2433014)

97 41 or 64 or 96 (3378282)

98 13 and 97 (190)

***************************

Database: Ovid MEDLINE(R) ALL <1946 to May 31, 2022>

Search Strategy:

--------------------------------------------------------------------------------

1 exp Rhinitis, Allergic/ (22963)

2 ((seasonal or intermittent or persistent or perennial or ragweed or grass or birch or house dust mite or mold or dander or cat or aeroallergen) adj4 (allergic rhinitis or allergic rhinitides)).mp. (3669)

3 hay fever.mp. (3878)

4 1 or 2 or 3 (24801)

5 Sublingual Immunotherapy/ (654)

6 ((SLIT or sublingual immunotherap*) adj2 tablet*).mp. (213)

7 5 or 6 (741)

8 Subcutaneous immunotherap*.mp. (994)

9 SCIT.mp. (774)

10 8 or 9 (1288)

11 7 or 10 (1855)

12 4 and 11 (841)

13 limit 12 to (english language and humans and yr="2016 -Current") (372)

14 (((Three or "3") adj year*) or 36 month*).mp. (236652)

15 (((Five or "5") adj year*) or 60 month*).mp. (364167)

16 (((Four or "4") adj year*) or 48 month*).mp. (134004)

17 (Long adj term).mp. (927154)

18 (Multiyear or multi-year).mp. (3058)

19 (("36" or "48" or "60") adj month*).mp. (53456)

20 or/14-19 (1530168)

21 13 and 20 (91)

***************************

## Cochrane Databases Search Strategy (May 19, 2022)

S1 Allergic Rhinitis

S2 ((seasonal or intermittent or persistent or perennial or ragweed or grass or birch or house dust mite or mold or dander or cat or aeroallergen) n4 (allergic rhinitis or allergic rhinitides))

S3 hay fever

S4 S1 OR S2 OR S3

S5 (SLIT or sublingual immunotherap*)

S6 (SCIT or Subcutaneous immunotherap*)

S7 S5 OR S6

S8 S4 AND S7 (3)

## Internet Search Strategies

| Date | Repository | Search terms | # Results |
| --- | --- | --- | --- |
| May 24, 2022 | Google (first 5 pages) | - allergic rhinitis AND (SCIT OR SLIT) - allergic rhinitis AND (Sublingual Immunotherapy OR Subcutaneous immunotherapy) - allergic rhinitis AND (Sublingual Immunotherapy OR Subcutaneous immunotherapy) AND (3 years or 4 years or 5 years or multiyear or long term or 36 months or 48 months or 60 months) - allergic rhinitis AND (SLIT OR SCIT) AND (3 years or 4 years or 5 years or multiyear or long term or 36 months or 48 months or 60 months) | 118 |
| May 24, 2022 | CMA Infobase | - Sublingual Immunotherapy - Subcutaneous immunotherapy - SLIT - SCIT | 0 |
| May 24, 2022 | NICE | - Sublingual Immunotherapy - Subcutaneous immunotherapy - SLIT - SCIT | 17 |
| May 24, 2022 | Health Canada | - Sublingual Immunotherapy - Subcutaneous immunotherapy - SLIT - SCIT | 93 |
| May 24, 2022 | National Institutes of Health | - Sublingual Immunotherapy AND allergic rhinitis - Subcutaneous immunotherapy AND allergic rhinitis | 100 |
| May 24, 2022 | European Academy of Allergy and Clinical Immunology | Via Google: "sublingual immunotherapy” site:<https://www.eaaci.org/>  "subcutaneous immunotherapy" site:<https://www.eaaci.org/> | 11 |
| May 24, 2022 | Australasian Society of Clinical Immunology and Allergy | Via Google: "subcutaneous immunotherapy" site:<https://www.allergy.org.au/>  "subcutaneous immunotherapy" site:<https://www.allergy.org.au/> | 80 |
| May 24, 2022 | American Academy of Allergy, Asthma & Immunology | Via Google: "sublingual immunotherapy” site:<https://www.aaaai.org/>  "subcutaneous immunotherapy” site:<https://www.aaaai.org/> | 100 |

# Appendix 6

## Research Question 6: Based on efficacy data, should ALL patients seen by an allergist be offered SLIT or SCIT as a treatment option?

## Inclusion/Exclusion Criteria

A total of 193 results were returned in the database searches and 501 results were returned from searching the Internet and online resources. A reviewer screened these results for relevance to the research question and applied the inclusion and exclusion criteria for this project. Duplicate results from different searches were removed. The inclusion criteria were as follows:

- Adult or pediatric participants with allergic rhinitis
- Treatment with SCIT or SLIT tablets
- Clinical practice guidelines, systematic reviews or meta-analyses, or randomized controlled trials were prioritized
- Clinical practice guidelines, systematic reviews or randomized controlled trials of all treatment lengths
- Papers such as systematic reviews that included data from treatment with SLIT drops as well as SLIT tablets were included with an identifying note.
- English language
- Published in the year 2016 or later
- Human subject studies

Exclusion criteria for the search was as follows:

- Studies focused on treatment with only SLIT drops and not tablets
- Articles focused on food allergy and not environmental allergens
- Articles focused on local allergic rhinitis, non-allergic rhinitis, occupational allergic rhinitis or other respiratory conditions such as asthma, that did not also include allergic rhinitis patients.

## Medline Search Strategies

Database: Ovid MEDLINE(R) ALL <1946 to May 18, 2022>

Search Strategy:

--------------------------------------------------------------------------------

1 exp Rhinitis, Allergic/ (22940)

2 ((seasonal or intermittent or persistent or perennial or ragweed or grass or birch or house dust mite or mold or dander or cat or aeroallergen) adj4 (allergic rhinitis or allergic rhinitides)).mp. (3667)

3 hay fever.mp. (3875)

4 1 or 2 or 3 (24775)

5 Sublingual Immunotherapy/ (654)

6 ((SLIT or sublingual immunotherap*) adj2 tablet*).mp. (213)

7 5 or 6 (741)

8 Subcutaneous immunotherap*.mp. (992)

9 SCIT.mp. (773)

10 8 or 9 (1286)

11 7 or 10 (1853)

12 4 and 11 (840)

13 limit 12 to (english language and humans and yr="2016 -Current") (371)

14 exp clinical pathway/ (7500)

15 exp clinical protocol/ (184661)

16 clinical protocols/ (29675)

17 exp consensus/ (18497)

18 exp consensus development conference/ (12611)

19 exp consensus development conferences as topic/ (2996)

20 critical pathways/ (7500)

21 exp guideline/ (37033)

22 guidelines as topic/ (42001)

23 exp practice guideline/ (29828)

24 practice guidelines as topic/ (127285)

25 health planning guidelines/ (4164)

26 Clinical Decision Rules/ (868)

27 (guideline or practice guideline or consensus development conference or consensus development conference, NIH).pt. (46885)

28 (position statement* or policy statement* or practice parameter* or best practice*).ti,ab,kf,kw. (41432)

29 (standards or guideline or guidelines).ti,kf,kw. (126495)

30 ((practice or treatment* or clinical) adj guideline*).ab. (48059)

31 (CPG or CPGs).ti. (6195)

32 consensus*.ti,kf,kw. (31591)

33 consensus*.ab. /freq=2 {Including Related Terms} (1)

34 ((critical or clinical or practice) adj2 (path or paths or pathway or pathways or protocol*)).ti,ab,kf,kw. (24274)

35 recommendat*.ti,kf,kw. or guideline recommendation*.ab. (53494)

36 (care adj2 (standard or path or paths or pathway or pathways or map or maps or plan or plans)).ti,ab,kf,kw. (74313)

37 (algorithm* adj2 (screening or examination or test or tested or testing or assessment* or diagnosis or diagnoses or diagnosed or diagnosing)).ti,ab,kf,kw. (9343)

38 (algorithm* adj2 (pharmacotherap* or chemotherap* or chemotreatment* or therap* or treatment* or intervention*)).ti,ab,kf,kw. (11861)

39 (guideline* or standards or consensus* or recommendat*).au. (553)

40 (guideline* or standards or consensus* or recommendat*).ca. (1187)

41 or/14-40 (691533)

42 (systematic review or meta-analysis).pt. (272085)

43 meta-analysis/ or systematic review/ or systematic reviews as topic/ or meta-analysis as topic/ or "meta analysis (topic)"/ or "systematic review (topic)"/ or exp technology assessment, biomedical/ or network meta-analysis/ (307857)

44 ((systematic* adj3 (review* or overview*)) or (methodologic* adj3 (review* or overview*))).ti,ab,kf,kw. (269179)

45 ((quantitative adj3 (review* or overview* or synthes*)) or (research adj3 (integrati* or overview*))).ti,ab,kf,kw. (13759)

46 ((integrative adj3 (review* or overview*)) or (collaborative adj3 (review* or overview*)) or (pool* adj3 analy*)).ti,ab,kf,kw. (34377)

47 (data synthes* or data extraction* or data abstraction*).ti,ab,kf,kw. (35145)

48 (handsearch* or hand search*).ti,ab,kf,kw. (10461)

49 (mantel haenszel or peto or der simonian or dersimonian or fixed effect* or latin square*).ti,ab,kf,kw. (31979)

50 (met analy* or metanaly* or technology assessment* or HTA or HTAs or technology overview* or technology appraisal*).ti,ab,kf,kw. (11109)

51 (meta regression* or metaregression*).ti,ab,kf,kw. (12429)

52 (meta-analy* or metaanaly* or systematic review* or biomedical technology assessment* or bio-medical technology assessment*).mp,hw. (405527)

53 (medline or cochrane or pubmed or medlars or embase or cinahl).ti,ab,hw. (294500)

54 (cochrane or (health adj2 technology assessment) or evidence report).jw. (20744)

55 (comparative adj3 (efficacy or effectiveness)).ti,ab,kf,kw. (16004)

56 (outcomes research or relative effectiveness).ti,ab,kf,kw. (10586)

57 ((indirect or indirect treatment or mixed-treatment or bayesian) adj3 comparison*).ti,ab,kf,kw. (3939)

58 (multi* adj3 treatment adj3 comparison*).ti,ab,kf,kw. (275)

59 (mixed adj3 treatment adj3 (meta-analy* or metaanaly*)).ti,ab,kf,kw. (174)

60 umbrella review*.ti,ab,kf,kw. (962)

61 (multi* adj2 paramet* adj2 evidence adj2 synthesis).ti,ab,kw,kf. (13)

62 (multiparamet* adj2 evidence adj2 synthesis).ti,ab,kw,kf. (17)

63 (multi-paramet* adj2 evidence adj2 synthesis).ti,ab,kw,kf. (11)

64 or/42-63 (600606)

65 (Randomized Controlled Trial or Controlled Clinical Trial or Pragmatic Clinical Trial or Equivalence Trial or Clinical Trial, Phase III).pt. (662932)

66 Randomized Controlled Trial/ (568356)

67 exp Randomized Controlled Trials as Topic/ (159145)

68 "Randomized Controlled Trial (topic)"/ (0)

69 Controlled Clinical Trial/ (94874)

70 exp Controlled Clinical Trials as Topic/ (164824)

71 "Controlled Clinical Trial (topic)"/ (0)

72 Randomization/ (106844)

73 Random Allocation/ (106844)

74 Double-Blind Method/ (171696)

75 Double Blind Procedure/ (0)

76 Double-Blind Studies/ (171696)

77 Single-Blind Method/ (31921)

78 Single Blind Procedure/ (0)

79 Single-Blind Studies/ (31921)

80 Placebos/ (35915)

81 Placebo/ (0)

82 Control Groups/ (1830)

83 Control Group/ (1830)

84 (random* or sham or placebo*).ti,ab,hw,kf,kw. (1695768)

85 ((singl* or doubl*) adj (blind* or dumm* or mask*)).ti,ab,hw,kf,kw. (257202)

86 ((tripl* or trebl*) adj (blind* or dumm* or mask*)).ti,ab,hw,kf,kw. (1371)

87 (control* adj3 (study or studies or trial* or group*)).ti,ab,kf,kw. (1133589)

88 (Nonrandom* or non random* or non-random* or quasi-random* or quasirandom*).ti,ab,hw,kf,kw. (50588)

89 allocated.ti,ab,hw. (77054)

90 ((open label or open-label) adj5 (study or studies or trial*)).ti,ab,hw,kf,kw. (40964)

91 ((equivalence or superiority or non-inferiority or noninferiority) adj3 (study or studies or trial*)).ti,ab,hw,kf,kw. (10714)

92 (pragmatic study or pragmatic studies).ti,ab,hw,kf,kw. (522)

93 ((pragmatic or practical) adj3 trial*).ti,ab,hw,kf,kw. (6898)

94 ((quasiexperimental or quasi-experimental) adj3 (study or studies or trial*)).ti,ab,hw,kf,kw. (10399)

95 (phase adj3 (III or "3") adj3 (study or studies or trial*)).ti,hw,kf,kw. (33313)

96 or/65-95 (2433014)

97 41 or 64 or 96 (3378282)

98 13 and 97 (190)

***************************

## Cochrane Databases Search Strategy (May 19, 2022)

S1 Allergic Rhinitis

S2 ((seasonal or intermittent or persistent or perennial or ragweed or grass or birch or house dust mite or mold or dander or cat or aeroallergen) n4 (allergic rhinitis or allergic rhinitides))

S3 hay fever

S4 S1 OR S2 OR S3

S5 (SLIT or sublingual immunotherap*)

S6 (SCIT or Subcutaneous immunotherap*)

S7 S5 OR S6

S8 S4 AND S7 (3)

## Internet Search Strategies

| Date | Repository | Search terms | # Results |
| --- | --- | --- | --- |
| May 24, 2022 | Google (first 5 pages) | - allergic rhinitis AND (SCIT OR SLIT) - allergic rhinitis AND (Sublingual Immunotherapy OR Subcutaneous immunotherapy) | 100 |
| May 24, 2022 | CMA Infobase | - Sublingual Immunotherapy - Subcutaneous immunotherapy - SLIT - SCIT | 0 |
| May 24, 2022 | NICE | - Sublingual Immunotherapy - Subcutaneous immunotherapy - SLIT - SCIT | 17 |
| May 24, 2022 | Health Canada | - Sublingual Immunotherapy - Subcutaneous immunotherapy - SLIT - SCIT | 93 |
| May 24, 2022 | National Institutes of Health | - Sublingual Immunotherapy AND allergic rhinitis - Subcutaneous immunotherapy AND allergic rhinitis | 100 |
| May 24, 2022 | European Academy of Allergy and Clinical Immunology | Via Google: "sublingual immunotherapy” site:<https://www.eaaci.org/>  "subcutaneous immunotherapy" site:<https://www.eaaci.org/> | 11 |
| May 24, 2022 | Australasian Society of Clinical Immunology and Allergy | Via Google: "subcutaneous immunotherapy" site:<https://www.allergy.org.au/>  "subcutaneous immunotherapy" site:<https://www.allergy.org.au/> | 80 |
| May 24, 2022 | American Academy of Allergy, Asthma & Immunology | Via Google: "sublingual immunotherapy” site:<https://www.aaaai.org/>  "subcutaneous immunotherapy” site:<https://www.aaaai.org/> | 100 |
